# The PAR2 inhibitor I-287 selectively targets Gα_q_ and Gα_12/13_ signaling and has anti-inflammatory effects

**DOI:** 10.1038/s42003-020-01453-8

**Published:** 2020-11-27

**Authors:** Charlotte Avet, Claudio Sturino, Sébastien Grastilleur, Christian Le Gouill, Meriem Semache, Florence Gross, Louis Gendron, Youssef Bennani, Joseph A. Mancini, Camil E. Sayegh, Michel Bouvier

**Affiliations:** 1 Institute for Research in Immunology and Cancer, and Department of Biochemistry and Molecular Medicine, Université de Montréal, Montréal, QC Canada H3C 1J4; 2 Vertex Pharmaceuticals (Canada), Inc., Laval, QC Canada H7V 4A7; 3 Département de Pharmacologie-Physiologie, Université de Sherbrooke, Centre de Recherche du CHU de Sherbrooke, Centre d’Excellence en Neurosciences de l’Université de Sherbrooke, Institut de Pharmacologie de Sherbrooke, Sherbrooke, QC Canada J1H 5N4; 4 Present Address: Paraza Pharma, Inc., Saint-Laurent, QC Canada H4S 2E1; 5 Present Address: Domain Therapeutics North America, Saint-Laurent, QC Canada H4S 1Z9; 6 Present Address: AdMare BioInnovations, Saint-Laurent, QC Canada H4S 1Z9; 7 Present Address: Vertex Pharmaceuticals Inc., Boston, MA 02210 USA; 8 Present Address: Ra Pharmaceuticals, Inc., Cambridge, MA 02140 USA

**Keywords:** Drug discovery, Cell biology

## Abstract

Protease-activated receptor-2 (PAR2) is involved in inflammatory responses and pain, therefore representing a promising therapeutic target for the treatment of immune-mediated inflammatory diseases. However, as for other GPCRs, PAR2 can activate multiple signaling pathways and those involved in inflammatory responses remain poorly defined. Here, we describe a new selective and potent PAR2 inhibitor (I-287) that shows functional selectivity by acting as a negative allosteric regulator on Gα_q_ and Gα_12/13_ activity and their downstream effectors, while having no effect on G_i/o_ signaling and βarrestin2 engagement. Such selective inhibition of only a subset of the pathways engaged by PAR2 was found to be sufficient to block inflammation in vivo. In addition to unraveling the PAR2 signaling pathways involved in the pro-inflammatory response, our study opens the path toward the development of new functionally selective drugs with reduced liabilities that could arise from blocking all the signaling activities controlled by the receptor.

## Abstract (web summary)

Avet et al. characterize I-287, an inhibitor to protease-activated receptor 2 using BRET-assays. They find that I-287 selectively inhibits Gα_q_ and Gα_12/13_ without affecting the activation of G_i/o_ or the recruitment of βarrestin2 and that it blocks inflammation in vitro and in vivo.

## Introduction

The protease-activated receptors (PARs) family, which comprises four members (PAR1–4), represents an atypical group among G-protein-coupled receptors (GPCRs), as they are activated by proteases rather than by the binding of soluble ligands. PARs are activated by the proteolytic cleavage of their N-terminal region by proteases such as thrombin, trypsin, and others. This cleavage exposes a region of the N-terminal extracellular domain, “tethered ligand” (TL), which then binds to the extracellular loops and transmembrane (TM) domains of the PARs. This results in the stabilization of an active conformation of the receptor that induces intracellular signal transduction^1,2^.

Among them, PAR2 is expressed in a wide range of cellular types, including endothelial, epithelial, immune, neuronal and smooth muscle cells, where it has been involved in multiple physiological and pathophysiological processes including nociception, cellular permeability, contractility, motility, proliferation, inflammatory responses, and cancers^1,2^. PAR2 is activated mainly by trypsin-like serine proteases such as trypsin, mast cell tryptase, tissue kallikreins, and coagulation factors VIIa and Xa^1,3–5^, but some proteases such as elastase or cathepsin-S have also been shown to cleave the receptor at non-canonical sites leading to a distinct activating sequence than the canonical TL and different signaling^6–8^. Short synthetic peptides mimicking the TL sequences, also called activating peptides, such as SLIGKV-NH_2_, SLIGRL-NH_2_, or 2-furoyl-LIGRL-NH_2_, can also directly activate PAR2 without proteolytic cleavage of its N-terminal extremity^9–11^. In addition, a subset of activating peptides (e.g., 2f-LAAAAI-NH_2_, Isox-Cha-Chg-NH_2_, and Isox-Cha-Chg-Ala-Arg-NH_2_) have been shown to promote the activation of only a subset of the signaling pathways engaged by PAR2; a concept known as functional selectivity or biased signaling^12^. PAR2 has been shown to activate a wide variety of intracellular signaling pathways, including G-protein-dependent pathways leading to Ca^2+^ mobilization, inhibition of cAMP production, mitogen-activated protein kinase ERK1/2 activation, Rho activation, and G-protein-independent signaling through βarrestin1/2 recruitment^1,2^.

Mounting evidence suggest that PAR2 plays an important role in mediating some of the inflammatory and pain responses associated with immune-mediated inflammatory diseases (IMIDs), a collection of highly disabling conditions resulting from the pathological activation of inflammatory pathways. PAR2 activation results in the release of inflammatory cytokine and chemokine from keratinocytes, endothelial, and epithelial cells^13^. Moreover, the administration of PAR2-activating proteases and synthetic agonists in vivo induce inflammatory responses^14–16^. Further, both in vitro and in vivo studies have demonstrated a role for PAR2 activation in tissue remodeling by promoting fibroblast and myofibroblast proliferation, and the secretion of growth factors such as connective tissue growth factor and extracellular matrix components including collagen^17^. In addition, PAR2 activation is implicated in cellular migration and has recently been shown to promote tumor growth and metastasis^18–20^. Finally, using PAR2-deficient mice or blocking receptor activation using specific antibodies or antagonists such as GB88 revealed an important role for PAR2 activation in the pathophysiology of a variety of IMID, including asthma, chronic pain, rheumatoid arthritis, periodontitis, inflammatory bowel diseases, skin diseases, cancer, fibrotic diseases, and neurological disease^21^. Although currently available PAR2 antagonists act through a variety of mechanisms that can be leveraged to understand the impact of PAR2 blockade, the specific pathway(s) mediating PAR2-dependent inflammatory effects remains poorly defined.

In the present study, we report the signaling properties of I-287, a bicyclic heteroaryl derivative developed by Vertex Pharmaceuticals and described in the patent WO2016154075^22^. Using bioluminescence resonance energy transfer (BRET)-based assays that allow direct monitoring of the engagement and activation of proximal signaling effectors (Supplementary Table 1), we first established the signaling repertoire of PAR2 triggered by trypsin and PAR2-activating peptides, and then established the functional selectivity of I-287 that leads to anti-inflammatory activity in vitro and in vivo. Taken together, our results demonstrated that blocking the activation of G_q_ and G_12/13_ without affecting the activation of G_i/o_ or the recruitment of β-arrestin is sufficient to blunt PAR2-mediated inflammatory responses.

## Results

### G-protein activation profile of PAR2

**Fig. 1:**
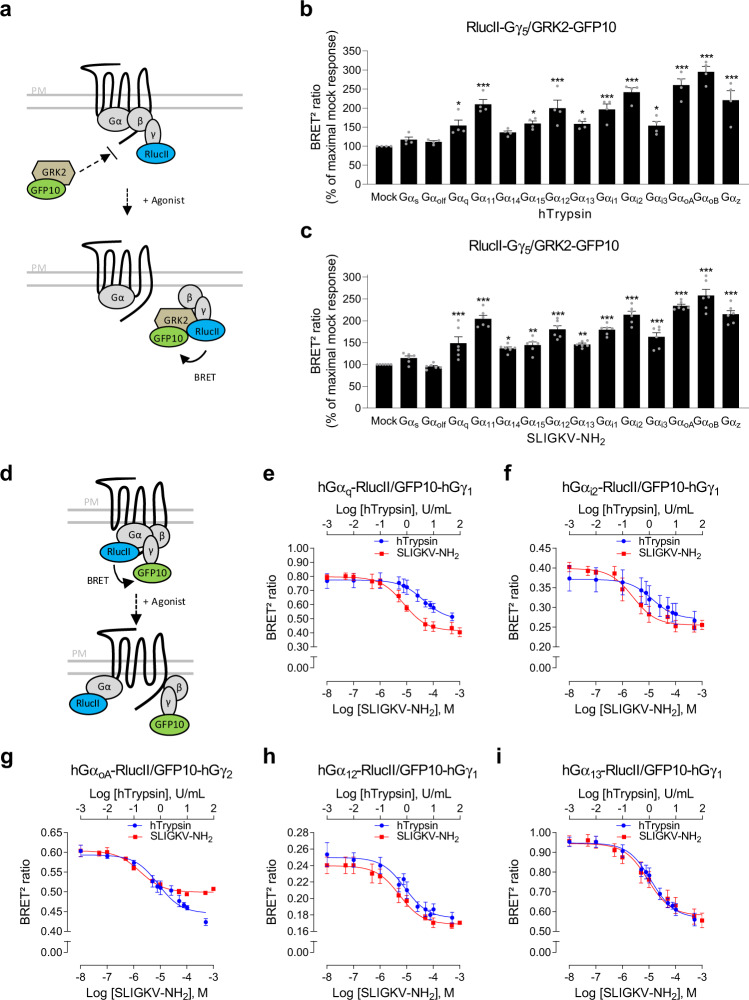
G-protein activation profile of hPAR2 in response to hTrypsin and SLIGKV-NH_2_ in HEK293 cells. **a** Schematic representation of the GRK-based BRET biosensor monitoring the activation of selective Gα subunits. Upon agonist stimulation, the Gα subunit dissociates from the βγ dimer (RlucII-Gγ_5_), allowing GRK2 sensor (GRK2-GFP10) recruitment to the βγ dimer and leading to an increase in BRET^2^ signal. **b**, **c** G-protein activation profiles induced by hTrypsin (10 U/mL, 15 min; **b**) or SLIGKV-NH_2_ (1 mM, 15 min; **c**) in HEK293 cells expressing hPAR2, and components of the G-protein activation sensor (RlucII-Gγ_5_, GRK2-GFP10, Gβ_1_, and the indicated Gα subunit). Results are expressed as BRET^2^ ratio in % of maximal response obtained in mock condition (mean ± SEM; *n* = 4–6; one-way ANOVA followed by Dunnett’s post hoc: *p < 0.05, **p < 0.01, and ***p < 0.001 compared to mock condition). **d** Schematic representation of the BRET^2^-based biosensor monitoring the agonist-promoted Gα and Gγ subunit separation. Upon agonist stimulation, the Gα subunit (Gα-RlucII) dissociates from the βγ dimer (GFP10-Gγ), leading to a decrease in BRET^2^ signal. **e**–**i** Dose–response curves of G-protein activation induced by increasing concentrations of hTrypsin or SLIGKV-NH_2_ (1 min) in HEK293 cells expressing hPAR2, Gβ1, and the BRET^2^-based α/βγ dissociation biosensors (GFP10-Gγ_1_ (**e**, **f**, **h**, **i**) or GFP10-Gγ_2_ (**g**) along with the indicated Gα-RlucII subunit). Results are expressed as BRET^2^ ratio of absolute values (mean ± SEM; *n* = 3).

As only a few numbers of studies have clearly demonstrated the direct coupling of PAR2 to specific Gα isoforms^23–25^, we first established the profile of Gα subunits activated by human PAR2 (hPAR2) using a BRET^2^-based assay platform in human embryonic kidney 293 (HEK293) cells. We took advantage of an assay based on the competition between GPCR kinase-2 (GRK2) and Gα for Gβγ, by measuring BRET between GRK2-GFP10 and RlucII-Gγ_5_ in the presence or absence of individual overexpressed Gα subunits (Fig. 1a)^26^. Cell treatment with human trypsin (hTrypsin) or SLIGKV-NH_2_ induced the activation of members of G_q/11_ (Gα_q_, Gα_11_, and Gα_15_) and G_i/o_ (Gα_i1_, Gα_i2_, Gα_i3_, Gα_oA_, Gα_oB_, and Gα_z_) families, as well as G_12/13_. In contrast, neither Gα_s_ nor Gα_olf_ were activated by hPAR2 in response to hTrypsin or SLIGKV-NH_2_ (Fig. 1b, c). This promiscuity of PAR2 coupling contrasts with the high selectivity of the M3-muscarinic acetylcholine receptor (M3-mAChR) used as a selectivity control and found to activate only G_q/11_ family members (Supplementary Fig. 1).

The activation of G-protein subtypes was further confirmed using a different BRET^2^-based assay. Contrary to the GRK-based sensor that was based on the competition between GRK protein and Gα subunit to bind Gβγ dimer, this assay directly monitors the separation of Gα fused to *Renilla* luciferase (Gα-RlucII) from Gγ fused to GFP10 (GFP10-Gγ) in living cells^26–28^ (Fig. 1d). Both hTrypsin and SLIGKV-NH_2_ promoted a rapid and concentration-dependent decrease in BRET^2^ for the human Gα_q_ (Fig. 1e), Gα_i2_ (Fig. 1f), Gα_oA_ (Fig. 1g), Gα_12_ (Fig. 1h), and Gα_13_ (Fig. 1i) sensors in HEK293 cells co-expressing hPAR2 (Supplementary Fig. 2a). Similar concentration–response curves were observed in HCT 116 cells co-expressing hPAR2 and the human biosensors (Supplementary Fig. 2b). The interspecies correspondence has been validated for G_q_ and G_i2_ using mouse sequence-based biosensors co-expressed with the mouse receptor (mPAR2) and stimulated by the mouse-selective activating peptide, SLIGRL-NH_2,_ in mouse rectal carcinoma CMT-93 cells (Supplementary Fig. 2c). A summary of potencies (pEC_50_) and maximal efficacies (*E*_max_) obtained are presented in Supplementary Fig. 2d.

We also compared the responses obtained with the Gα and Gγ subunits separation biosensors (Fig. 1e–i) to those generated with the GRK-based BRET biosensor (Fig. 1b, c) as *X*–*Y* plots (Supplementary Fig. 3). As can be seen in panel a, for each G protein an excellent linear correlation between the two sensors can be seen for all concentrations of hTrypsin. When comparing the maximal response for each G protein using the two sensors, again a straight relationship was observed (Supplementary Fig. 3b). However, the slope of the curve indicates that the two biosensors have slightly different sensitivities for the different G protein; the GRK2-based biosensor shows relatively greater sensitivity for G_i2_ and G_oA_, whereas the Gα and Gγ subunits separation sensor shows a greater sensitivity for G_q_ and G_13_. It should be noted that, as each biosensor comprised different components, the absolute BRET values do not allow to compare the “coupling strength” among G-protein subtypes but rather allow to determine whether a pathway is activated or not, and whether and how each of the responses can be modulated by different compounds.

### G-protein-dependent signaling pathways activated by PAR2

**Fig. 2:**
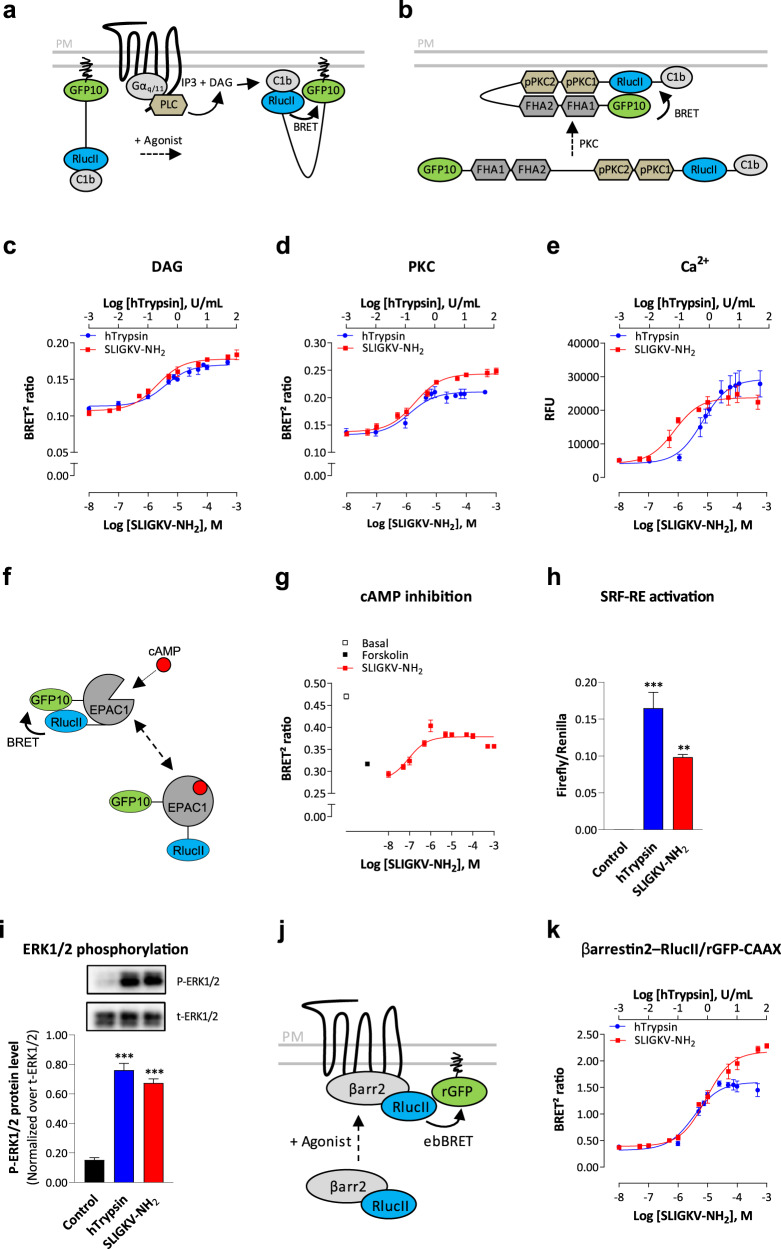
hPAR2 promotes signaling pathways downstream of Gα_q_, Gα_i/o_, and Gα_12/13_ proteins, and βarrestin2 recruitment at the plasma membrane in HEK293 cells. **a** Schematic representation of the unimolecular DAG BRET sensor, which measures the generation of DAG by activated PLC. The recruitment of c1b DAG-binding domain of PKCδ to the plasma membrane by DAG brings RlucII and GFP10 in close proximity, leading to an increase of BRET signal. **b** Schematic representation of the unimolecular PKC BRET sensor. The phosphorylation of PKC consensus sequences (pPKC1 and 2) induces their interaction with phosphothreonine-binding domains (FHA1 and FHA2) of Rad53 and allows a conformational change, leading to the close proximity of RlucII and GFP10 and an increased BRET signal. **c**, **d** Dose–response curves of DAG production (**c**) and PKC activation (**d**) induced by increasing concentrations of hTrypsin or SLIGKV-NH_2_ during 1 (DAG) or 5 min (PKC) in HEK293 cells expressing hPAR2 and the corresponding unimolecular BRET^2^-based biosensors. Results are expressed as BRET^2^ ratio of absolute values (mean ± SEM; *n* = 3). **e** Dose–response curves of Ca^2+^ mobilization (increases of peak values in relative fluorescence unit, RFU) induced by increasing concentrations of hTrypsin or SLIGKV-NH_2_ in HEK293 cells endogenously expressing hPAR2 (mean ± SEM; *n* = 5–7). **f** Schematic representation of the unimolecular EPAC BRET sensor, which measures the generation of cAMP by activated adenylate cyclase. cAMP binding to EPAC1 domain induces a conformational change, increasing the distance between RlucII and GFP10, and yielding to a reduction of the BRET signal . **g** Dose–response curves of hPAR2-mediated inhibition of forskolin-induced cAMP production in HEK293 cells expressing hPAR2. Cells expressing EPAC biosensor were stimulated during 5 min with increasing concentrations of SLIGKV-NH_2_ and then treated with forskolin (1.5 µM, 5 min) before measurement of cAMP production. Results are expressed as BRET^2^ ratio of absolute values (mean ± SEM; *n* = 3). **h** hPAR2-mediated activation of SRF-RE reporter gene, reflecting RhoA activation, induced by hTrypsin (10 U/mL, 6 h) or SLIGKV-NH_2_ (100 µM, 6 h) in HEK293 cells expressing hPAR2. Results are expressed as a ratio of Firefly over *Renilla* luminescence (mean ± SEM; *n* = 3; one-way ANOVA: **p < 0.01 and ***p < 0.001 compared to control cells). **i** ERK1/2 phosphorylation in HEK293 cells expressing hPAR2 and stimulated with hTrypsin (1 U/mL) or SLIGKV-NH_2_ (100 µM) for 10 min. Representative immunoblots of ERK1/2 phosphorylation are shown. Western blottings were quantified and expressed as the ratio of phosphorylated ERK (P-ERK1/2) protein level normalized over total ERK (t-ERK1/2) protein (mean ± SEM; *n* = 4; one-way ANOVA: ***p < 0.001 compared to control cells). **j** Schematic representation of the ebBRET-based assay that monitors energy transfer between βarrestin2–RlucII and rGFP-CAAX targeted to the plasma membrane. **k** Dose–response curves of βarrestin2 recruitment induced by increasing concentrations of hTrypsin or SLIGKV-NH_2_ (15 min) in HEK293 cells expressing hPAR2 and ebBRET sensors βarrestin2–RlucII/rGFP-CAAX. Results are expressed as BRET^2^ ratio of absolute values (mean ± SEM; *n* = 3–4).

To further characterize the signaling profile of hPAR2, we evaluated its ability to activate downstream effectors of G proteins. We first evaluated the coupling of hPAR2 to signaling pathways downstream of G_q_ protein. In agreement with the results observed for G_q_ activation, both agonists promoted the production of diacylglycerol (DAG; Fig. 2c and Supplementary Fig. 4a, left panel) and protein kinase C (PKC; Fig. 2d and Supplementary Fig. 4a, central panel) activation as assessed by unimolecular BRET^2^-based biosensors (Fig. 2a, b)^29^. Ca^2+^ mobilization was also stimulated, as detected by the FLIPR-5 fluorescent dye, upon activation of the receptor (Fig. 2e and Supplementary Fig. 4a, right panel). Ca^2+^ release induced by hTrypsin or SLIGKV-NH_2_ was completely blocked by pretreatment with the specific G_q/11_ inhibitor YM-254890^30^, but not by the G_i/o_-specific inhibitor pertussis toxin (PTX) (Supplementary Fig. 4b, left panel), indicating that intracellular Ca^2+^ release is mediated through the coupling of hPAR2 to G_q/11_ but not G_i/o_. We also evaluated the role of G_12/13_ in the Ca^2+^ response using HEK293 cells genetically deleted for Gα_12_ and Gα_13_ proteins by the CRISPR/Cas9 system (ΔG_12/13_)^29,31^. G_12/13_ deletion, as well as reintroduction of either Gα_12_ (ΔG_12/13_: +G_12_) or Gα_13_ (ΔG_12/13_: +G_13_) into the KO background, had no impact on the Ca^2+^ response (Supplementary Fig. 4b, right panel), demonstrating that G_12/13_ were not required for hPAR2-mediated Ca^2+^ mobilization. Similar productions of DAG and Ca^2+^, as well as PKC activation were observed following hPAR2 activation in HCT 116 cells and mPAR2 activation by SLIGRL-NH_2_ in CMT-93 cells (Supplementary Fig. 4c, d, respectively). A summary of potencies (pEC_50_) and maximal efficacy (*E*_max_) values obtained are presented in Supplementary Fig. 4e.

To evaluate the regulation of adenylate cyclase by hPAR2-activated G_i/o_, we measured the inhibition of forskolin-induced cAMP production using the EPAC BRET^2^-based biosensor (Fig. 2f)^32^. The forskolin-promoted increase in cAMP level was concentration-dependently blocked by hPAR2 stimulation with SLIGKV-NH_2_ (Fig. 2g), illustrated by the increase of BRET^2^ signal compared to forskolin treated cells.

We then evaluated the G_12/13_ protein-dependent activation of RhoA by measuring the induction of serum response factor response element (SRF-RE) reporter gene^33^. Treatment of HEK293 cells with both PAR2 agonists induced SRF-RE gene (Fig. 2h), mainly through the G_12/13_ proteins since it was not inhibited by the G_q/11_ inhibitor YM-254890 in wild-type cells (Supplementary Fig. 5a). However, in cells lacking G_12/13_, the response became G_q_-dependent, as addition of YM-254890 in these cells blocked the response (Supplementary Fig. 5b). Reintroduction of either Gα_12_ or Gα_13_ into the G_12/13_ KO cells potentiated the hTrypsin and SLIGKV-NH_2_-promoted responses, while abolishing the YM-254890 effect (Supplementary Fig. 5b). This indicates that, in the absence of G_12/13_, the G_q_ coupling can support the SRF-RE activation but that its involvement is minimal when G_12/13_ are present.

Finally, we showed that hPAR2 activation leads to ERK1/2 phosphorylation by both agonists (Fig. 2i) through the activation of G_i/o_ and G_q_/PKC, as it was partially blocked by both the PTX and YM-254890 or the PKC inhibitor Gö 6983 (Supplementary Fig. 6a, b). In contrast, RhoA activation did not contribute to PAR2-mediated activation of ERK1/2, as the specific RhoA inhibitor, CT04, did not affect ERK phosphorylation (Supplementary Fig. 6c). Similarly, β-arrestin engagement was not essential for this activation, as it was also observed in HEK293 cells genetically deleted from βarrestin1/2 by the CRISPR/Cas9 system (Δβarrestin1/2; Supplementary Fig. 6d).

### βarrestin2 recruitment induced by PAR2 activation

We characterized the recruitment of βarrestin2 at the plasma membrane upon hPAR2 stimulation using enhanced bystander BRET (ebBRET) monitoring energy transfer between βarrestin2–RlucII and the plasma membrane-targeted rGFP-CAAX (Fig. 2j)^34^. Both hTrypsin and SLIGKV-NH_2_ induced concentration-dependent recruitment of βarrestin2 at the plasma membrane by hPAR2 in HEK293 cells (Fig. 2k and Supplementary Fig. 7a) and in HCT 116 cells, or by mPAR2 in CMT-93 in response to SLIGRL-NH_2_ (Supplementary Fig. 7b, c, respectively). A summary of potencies (pEC_50_) and maximal efficacies (*E*_max_) obtained are presented in Supplementary Fig. 7d.

Interestingly, as recently reported^35^, the synthetic peptide was found to be more efficacious than hTrypsin to promote the recruitment of β-arrestin. Similar observations were made for G_q_ and G_i2_ but not for G_oA_ or G_12_/G_13_ (Fig. 1e–i and Supplementary Fig. 2). It is unlikely that such difference results from an action on a different GPCR, because, as seen in Supplementary Fig. 7e, no βarrestin2 recruitment could be observed in either parental or KO cells lacking hPAR2 for either SLIGKV-NH_2_ or hTrypsin. The recruitment of βarrestin2 required the heterologous expression of hPAR2 in both cases. The most likely hypothesis is that SLIGKV-NH_2_ displays functional selectivity compared to hTrypsin favoring β-arrestin, G_q_ and G_i2_ over G_12/13_. Mechanistically, this would mean that the synthetic peptide would stabilize a conformation that is somewhat different from the one promoted by the TL, and that the former would be more favorable to βarrestin, G_q_ and G_i2_ engagement.

### Characterization of I-287 as a new PAR2 inhibitor

**Fig. 3:**
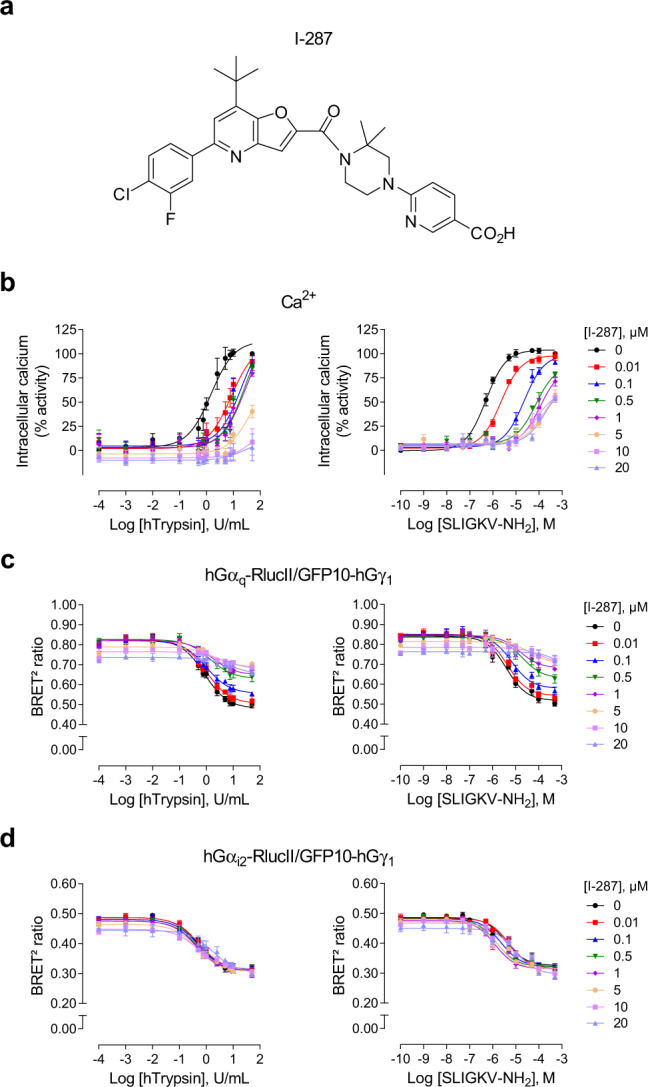
Identification of I-287 as a negative PAR2 allosteric modulator. **a** Chemical structure of compound I-287. **b** Impact of I-287 pretreatment (30 min) on the Ca^2+^ responses evoked by increasing concentrations of hTrypsin (left panel) or SLIGKV-NH_2_ (right panel) in HEK293 cells endogenously expressing hPAR2. Results are expressed as % of the maximal induced-response in the absence of I-287 (% activity; mean ± SEM; *n* = 3–6). **c**, **d** Impact of I-287 pretreatment (15 min) on the Gα_q_ (**c**) and Gα_i2_ (**d**) proteins activation induced after 1 min stimulation with increasing concentrations of hTrypsin (left panel) or SLIGKV-NH_2_ (right panel) in HEK293 cells co-expressing hPAR2 and the human BRET^2^-based biosensors Gα_q_-RlucII or Gα_i2_-RlucII and GFP10-Gγ_1_. Results are expressed as BRET^2^ ratio of absolute values (mean ± SEM; *n* = 3).

I-287 (Fig. 3a) was discovered by Vertex Pharmaceuticals as a potent inhibitor of PAR2, as part of a drug discovery research program aimed at identifying novel therapeutics^22^. To further characterize the properties of this compound, Schild analyses of the effects of I-287 on the trypsin- and SLIGKV-Ca^2+^ responses and on the activation of G_q_ and G_i2_ (through βγ), which are known to promote Ca^2+^ mobilization, were conducted. Concentration-dependent rightward shifts of the Ca^2+^ response (Fig. 3b) and G_q_ activation (Fig. 3c*)* stimulated by either agonist were observed. No such inhibitory action of I-287 was observed for PAR2-mediated G_i2_ activation (Fig. 3d). The G-protein subtype-selective inhibition observed for I-287 suggest an allosteric mode of action, as a competitive inhibitor would be expected to block the activation of both G-protein subtypes engaged by the receptor. Consistent with such an allosteric mode of action is the observation of both the rightward shifts collapse at the highest concentrations of the antagonist and the insurmountable aspect of the inhibition. This non-competitive mode of action strongly suggests that I-287 is a negative allosteric modulator (NAM) and not an orthosteric competitive antagonist of hPAR2.

### I-287 inhibits PAR2-mediated activation of G_q_ and G_12/13_ but not G_i/o_ proteins

**Fig. 4:**
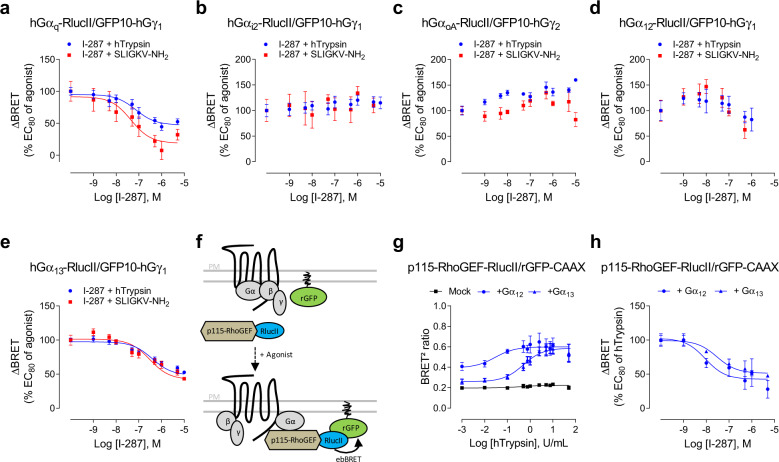
Biased effect of I-287 on hPAR2-promoted Gα protein activation. **a**–**e** Impact of I-287 on hPAR2-promoted G-protein activation measured by BRET^2^. HEK293 cells co-expressing hPAR2 and the human BRET^2^-based α/βγ dissociation biosensors (GFP10-Gγ_1_ or GFP10-Gγ_2_ along with the indicated Gα-RlucII subunit), were pretreated with increasing concentrations of I-287 for 15 min followed by 1 min stimulation with an EC_80_ concentration of hTrypsin or SLIGKV-NH_2_. Results are expressed as ΔBRET in % of the response induced by EC_80_ of respective agonists in the absence of I-287 (mean ± SEM; *n* = 3–6). **f** Schematic representation of the ebBRET-based biosensor to selectively monitor Gα_12/13_ activation. Upon agonist stimulation, activated Gα_12_ or Gα_13_ subunits recruit their selective effector p115-RhoGEF tagged with RlucII to the plasma membrane, leading to an increase of ebBRET with the membrane-anchored rGFP-CAAX. **g** Dose–response curves of p115-RhoGEF recruitment at the plasma membrane induced by increasing concentrations of hTrypsin for 1 min in HEK293 cells expressing hPAR2 and the p115-RhoGEF-RlucII/rGFP-CAAX sensors in the absence (mock) or in the presence of Gα_12_ or Gα_13_ subunits. Results are expressed as BRET^2^ ratio of absolute values (mean ± SEM; *n* = 4). **h** Inhibitory action of increasing concentrations of I-287 (15 min) on the G_12/13_-mediated recruitment of p115-RhoGEF at plasma membrane induced by an EC_80_ concentration of hTrypsin in HEK293 cells co-expressing hPAR2 and the human BRET^2^-based biosensors p115-RhoGEF-RlucII/rGFP-CAAX along with Gα_12_ or Gα_13_ subunits. Results are expressed as ΔBRET in % of the response induced by EC_80_ of hTrypsin in the absence of I-287 (mean ± SEM; *n* = 3).

Given the functional selectivity of I-287 toward G_q_ vs. G_i_, we further investigated the effect of this NAM on the activation of Gα_q_, Gα_i2_, Gα_oA_, Gα_12_, and Gα_13_ using the BRET^2^-based assays described previously. The concentration-dependent inhibition was tested using an EC_80_ concentration of agonist in HEK293 cells. The results, expressed as a % of the response induced by the agonists alone, show that I-287 inhibited both G_q_ and G_13_ (Fig. 4a, e) activation induced by either hTrypsin or SLIGKV-NH_2_ with IC_50_s between 45 and 390 nM (see Supplementary Fig. 8d), but was without effect on the activation of G_i2_ and G_oA_ (Fig. 4b, c). A similar G-protein subtype-selective effect of I-287 was observed in HCT 116 cells expressing hPAR2 (Supplementary Fig. 8a, d), and mPAR2 signaling in CMT-93 cells (Supplementary Fig. 8c, d), thus illustrating the inter-cell lines and the interspecies preservation of I-287 pathway-selective inhibitory effect on PAR2 signaling.

**Fig. 5:**
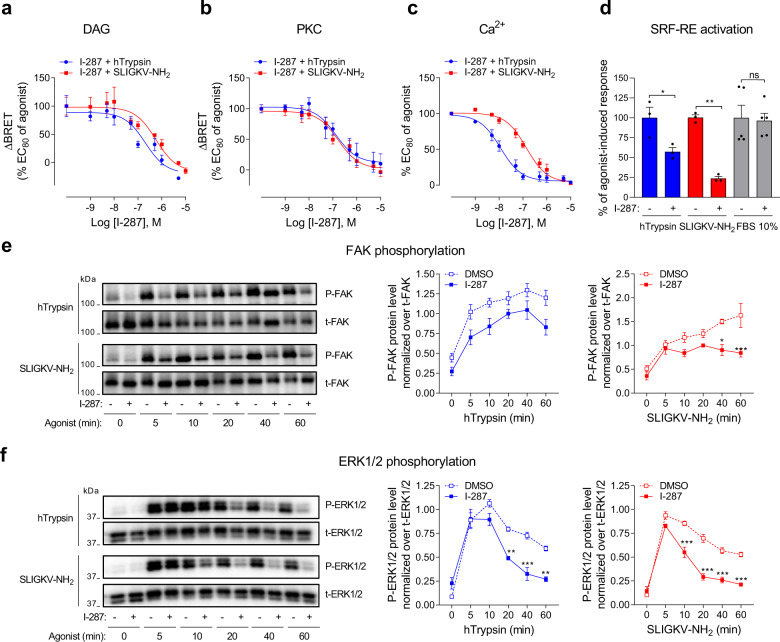
I-287 inhibits PAR2-mediated activation of DAG/Ca^2+^/PKC and RhoA/SRF-RE, as well as FAK and ERK1/2 signaling pathways. **a**, **b** Impact of increasing concentrations of I-287 (15 min) on DAG production (**a**) and PKC activation (**b**) induced after 1 (DAG) or 5 (PKC) min stimulation with an EC_80_ concentration of hTrypsin or SLIGKV-NH_2_ in HEK293 cells co-expressing hPAR2 and the indicated unimolecular BRET^2^-based biosensors. Results are expressed as ΔBRET in % of the response induced by EC_80_ of respective agonists in the absence of I-287 (mean ± SEM; *n* = 4–5). **c** Impact of increasing concentrations of I-287 (30 min) on intracellular Ca^2+^ mobilization induced by an EC_80_ concentration of hTrypsin or SLIGKV-NH_2_ in HEK293 cells endogenously expressing hPAR2. Results are expressed as % of the response induced by respective agonists in the absence of I-287 (mean ± SEM; *n* = 3-4). **d** Impact of I-287 (10 µM, 30 min) on hPAR2-promoted SRF-RE reporter gene activation induced after 6 h stimulation with hTrypsin (10 U/mL) or SLIGKV-NH_2_ (100 µM) in HEK293 cells expressing hPAR2. FBS (10%) was used as control. Results are expressed as % of the response induced by respective agonists in the absence of I-287 (mean ± SEM; *n* = 3–5; unpaired *t*-test: *p < 0.05 and **p < 0.01 compared to respective control cells, ns: nonsignificant). **e**, **f** Kinetics of FAK and ERK1/2 phosphorylation in HEK293 cells expressing hPAR2 and pretreated with DMSO or I-287 (10 µM, 30 min) before stimulation with hTrypsin (1 U/mL) or SLIGKV-NH_2_ (100 µM) at the indicated times. Representative immunoblots of FAK and ERK1/2 phosphorylation are shown. Western blots were quantified and expressed as the ratio of phosphorylated protein level (P-FAK or P-ERK1/2) normalized over total protein (t-FAK or t-ERK1/2; mean ± SEM; *n* = 3–5; two-way ANOVA followed by Tukey’s post hoc test: *p < 0.05, **p < 0.01, and ***p < 0.001 compared to DMSO-treated cells at the respective time).

In contrast to the clear inhibition observed for G_13_ (Fig. 4e), for G_12_, the effect of I-287 observed in both HEK293 and HCT 116 cell lines was ambiguous (Fig. 4d and Supplementary Fig 8a). To further characterize the engagement of G_12/13_ by PAR2 and its inhibition by I-287, we used a new ebBRET-based assay that monitors the recruitment of the selective G_12/13_ effector, p115-RhoGEF, to the activated (GTP-bound) G proteins (Fig. 4f). hTrypsin promoted an increase in ebBRET signal between p115-RhoGEF-RlucII and rGFP-CAAX (Fig. 4g), which was not affected neither by YM-254890, nor PTX (Supplementary Fig. 9a, left and central panel). In addition, hTrypsin promoted an ebBRET increase in HEK293 cells genetically deleted for Gα_12_ and Gα_13_ proteins (ΔG_12/13_)^29,31^ only following reintroduction of either Gα_12_ (ΔG_12/13_: +G_12_) or Gα_13_ (ΔG_12/13_: +G_13_) (Supplementary Fig. 9a, right panel). Similar results were observed with the activation of the human thromboxane A2α receptor (hTPαR), a well-documented G_12/13_ activating receptor, by U46691 (Supplementary Fig. 9b), validating the selectivity of the sensor for the activation of the G_12/13_ protein family members. Using this p115-RhoGEF-RlucII/rGFP-CAAX biosensors, we confirmed that I-287 can inhibits the responses evoked by both G_12_ and G_13_ (Fig. 4h). Finally, the selectivity of I-287 toward PAR2 is illustrated by the fact that it did not affect the activation of Gα_q_ induced by carbachol stimulation of M3-mAChR or Gα_12_ and Gα_13_ induced by U46691 stimulation of hTPαR (Supplementary Fig. 8b).

### I-287 inhibits PAR2-mediated activation of DAG/Ca^2+^/PKC, RhoA, SRF-RE, and FAK signaling pathways

We then examined the impact of I-287 on hPAR2-mediated activation of signaling pathways downstream of Gα_q_ and Gα_12/13_ by monitoring DAG/Ca^2+^/PKC and RhoA/FAK/SRF-RE pathways in HEK293 cells. Consistent with the ability of I-287 to inhibit G_q_, the NAM also blocked DAG production (Fig. 5a), PKC activation (Fig. 5b), as well as Ca^2+^ mobilization (Fig. 5c) with IC_50_s ranging from 10 to 500 nM depending of the pathway (see Supplementary Fig. 10e). A similar profile was observed in hPAR2-expressing HCT 116 and mPAR2-expressing CMT-93 cells, respectively (Supplementary Fig. 10a, b, e). In contrast, I-287 had no significant impact on DAG or PKC signaling pathways induced by carbachol stimulation of M3-mAChR (Supplementary Fig. 10c), confirming its selectivity for PAR2. However, an inhibitory action on the Ca^2+^ and PKC responses were observed but only at very high concentration (>1 µM) suggesting an off-target effect of the compound at very high concentration for these sensitive assay (Supplementary Fig. 10c). We cannot exclude the possibility of a shared allosteric site common to other G_q_ coupled receptors. However, no such effect was observed on M3R-mediated G_q_ activation measured directly (see Supplementary Fig. 8b).

Given that I-287 blocked Gα_12/13_ activation, we evaluated if the compound also affected downstream RhoA signaling by measuring the SRF-RE reporter gene induction in response to hPAR2 activation by hTrypsin or SLIGKV-NH_2_. In agreement with the effect observed on Gα_12/13_, pretreatment with I-287 significantly reduced the induction of SRF-RE reporter gene promoted by the two agonists (Fig. 5d). A similar inhibition was observed on SRF-RE gene induction mediated by hPAR2 stimulation by both agonists in HCT 116 cells (Supplementary Fig. 10d). This effect is selective for hPAR2, as I-287 had no effect on SRF-RE induction mediated by 10% fetal bovine serum (FBS), a known activator of RhoA/ROCK pathway, in HEK293 (Fig. 5d) and HCT 116 (Supplementary Fig. 10d) cells. Finally, we determined whether the inhibition of Gα_q_ and Gα_12/13_ could impact the activity of a protein involved in cytoskeletal reorganization, the focal adhesion kinase (FAK), known to be regulated by this axis^36^. As shown in Fig. 5e, I-287 significantly inhibited the SLIGKV-NH_2_-induced FAK phosphorylation after 40 and 60 min of agonist stimulation. Although a similar tendency was observed for the hTrypsin promoted response, the inhibition did not reach statistical significance.

### I-287 inhibits PAR2-m**e**diated ERK1/2 activation

We then assessed the impact of I-287 on ligand-promoted activation of ERK1/2 in HEK293 cells expressing hPAR2. Pretreatment of cells with I-287 significantly inhibited the time-dependent activation of ERK1/2 induced by hTrypsin and SLIGKV-NH_2_ (Fig. 5f). Given that we showed that PAR2-induced ERK1/2 activation is mediated by G_q/11_ and G_i/o_ proteins (Supplementary Fig. 6), our results strongly suggest that I-287 mediated its action on ERK1/2 through the inhibition of G_q_/DAG/ Ca^2+^/PKC cascade.

### I-287 has no effect on PAR2-mediated recruitment of βarrestin2 and receptor internalization

**Fig. 6:**
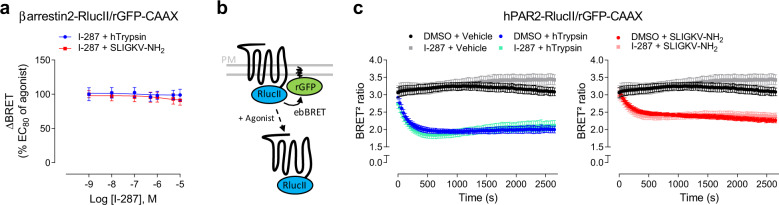
I-287 has no effect on βarrestin2 recruitment and PAR2 internalization. **a** Impact of increasing concentrations of I-287 (15 min) on βarrestin2 recruitment at the plasma membrane induced by an EC_80_ concentration of hTrypsin or SLIGKV-NH_2_ (15 min) in HEK293 cells co-expressing hPAR2 and the ebBRET sensors βarrestin2–RlucII/rGFP-CAAX. Results are expressed as ΔBRET in % of the response induced by EC_80_ of respective agonists in the absence of I-287 (mean ± SEM; *n* = 5). **b** Schematic representation of receptor internalization BRET-based biosensor using the hPAR2-RlucII and rGFP-CAAX sensors to monitor loss of hPAR2 from cell surface. **c** Impact of I-287 (1 µM, 15 min) on hPAR2 internalization kinetics induced by an EC_80_ concentration of hTrypsin or SLIGKV-NH_2_ in HEK293 cells expressing the hPAR2-RlucII/rGFP-CAAX sensors. Results are expressed as BRET^2^ ratio of absolute values (mean ± SEM; *n* = 3).

We then evaluated the impact of I-287 on hPAR2-mediated recruitment of βarrestin2 at the plasma membrane. I-287 did not affect the ebBRET signal between βarrestin2–RlucII and rGFP-CAAX promoted by both agonist stimulation of hPAR2 in either HEK293 (Fig. 6a) or HCT 116 cells, nor in mPAR2-expressing CMT-93 cells (Supplementary Fig. 10a, b, respectively).

We next sought to determine whether I-287 could affect the ligand-promoted endocytosis of hPAR2. For this purpose, we monitored ebBRET between *Renilla* luciferase-tagged receptor (hPAR2-RlucII) and the plasma membrane-anchored rGFP-CAAX (Fig. 6b). Both PAR2 agonists induced a reduction in BRET signal reflecting the loss of cell surface receptor resulting from its endocytosis. Pretreatment of cells with I-287 had no significant impact on neither the half-time nor on the maximal internalization induced by both agonists (Fig. 6c).

### I-287 inhibits PAR2-mediated IL-8 cytokine release

**Fig. 7:**
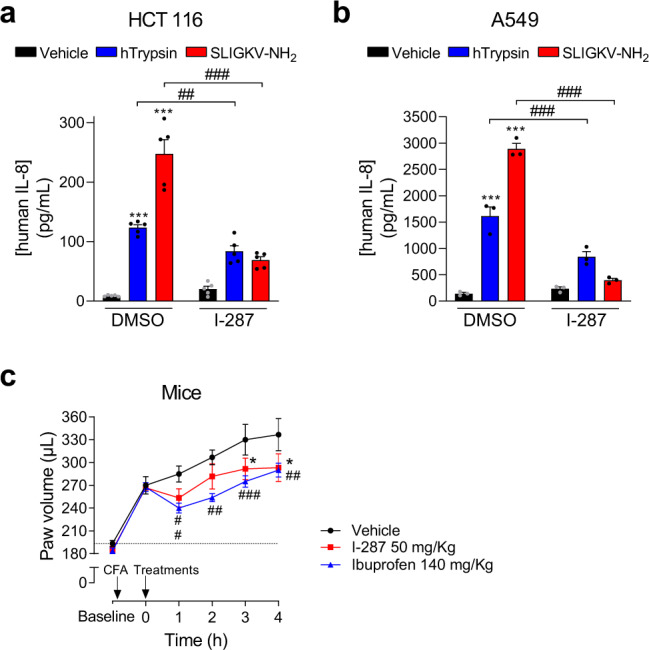
I-287 inhibits PAR2-induced secretion of IL-8 cytokine in vitro and reduces CFA-induced inflammation in mice. **a**, **b** Impact of I-287 (10 µM, 30 min) on hPAR2-promoted IL-8 cytokine release induced after 6 h stimulation with vehicle, hTrypsin (1 U/mL) or SLIGKV-NH_2_ (100 µM) in culture medium of HCT 116 (**a**) and A549 (**b**) cells expressing hPAR2. Data are expressed as IL-8 concentration in pg/mL (mean ± SEM; *n* = 3–5; two-way ANOVA followed by Tukey’s post hoc test: ***p < 0.001 compared to control cells with vehicle; ^##^*p* < 0.01 and ^###^*p* < 0.001 compared to control cells with respective agonist). **c** Impact of I-287 on complete Freund’s adjuvant (CFA)-induced inflammation in mice. One hour after CFA injection, mice were given I-287 (50 mg/kg) or vehicle (95% TPGS – 5% NMP) by gavage. A group of animals received Ibuprofen (140 mg/kg) as a reference drug. The volume of the hindpaw was measured every hour to evaluate swelling/inflammation using a plethysmometer (mean ± SEM; *n* = 6 for vehicle and I-287 groups and *n* = 8 for Ibuprofen group; two-way repeated-measures ANOVA followed by Dunnett’s post hoc test: *p < 0.05 for I-287 vs. vehicle and ^##^*p* < 0.01, ^###^*p* < 0001 for Ibuprofen vs. vehicle).

To determine whether PAR2 inhibition by I-287 may have anti-inflammatory effects, we assessed its ability to block the PAR2-mediated release of the inflammatory cytokine interleukin-8 (IL-8) in HCT 116 and human lung carcinoma (A549) cells. As shown in Fig. 7a, b, I-287 significantly blocked IL-8 secretion promoted by both hTrypsin and SLIGKV-NH_2_ in the two cell lines. No such inhibitory action of I-287 was observed on the tumor necrosis factor-α (TNF-α)-stimulated IL-8 secretion (Supplementary Fig. 11), supporting the selectivity of action. Surprisingly, I-287 pretreatment potentiated the IL-8 secretion elicited by TNF-α in HCT 116 cells (Supplementary Fig. 11a). This unexpected effect was not observed in A549 cells (Supplementary Fig. 11b), suggesting a cell-type-specific interaction between PAR2 and TNF-α pathways.

### I-287 reduces CFA-induced inflammation in mice

The in vivo anti-inflammatory properties of I-287 were then evaluated in the complete Freund’s adjuvant (CFA)-induced paw edema model in mice. CFA induced a time-dependent increase of the paw volume that was significantly reduced by oral administration (50 mg/kg) of I-287 3 h following the CFA treatment (Fig. 7c). This inhibitory action of I-287 was comparable to that of Ibuprofen (140 mg/kg), used as positive control. These results demonstrate that the functionally selective PAR2 antagonist I-287 is an orally active compound displaying an anti-inflammatory efficacy equivalent to one of the most used nonsteroidal anti-inflammatory drugs (NSAIDS) in the mice paw edema model. The good bioavailability of this compound measured in rats (58% bioavailability)^22^, suggests that I-287 is a good tool compound to study the anti-inflammatory actions of PAR2. Despite the fact that potentiating effect of I-287 on the TNF-α-promoted IL-8 secretion appears to be cell specific this potential caveat should be considered in studying its anti-inflammatory effectiveness.

## Discussion

Although PAR2 has been implicated in the pathogenesis of several IMIDs and cancers^2,37,38^, only a limited number of compounds able to block its action are currently available. The present study characterized I-287, a new PAR2-selective NAM that displays functional selectivity by inhibiting trypsin- and SLIGKV-NH_2_-mediated activation of G_q_ and G_12/13_ and their downstream signaling pathways without affecting G_i/o_ activation, nor affecting βarrestin2 recruitment and the resulting PAR2 internalization. Given the inhibitory action of I-287 on IL-8 secretion and on CFA-induced inflammatory response, the data demonstrate that blocking the G_q_ and G_12/13_ pathways is sufficient to promote PAR2-mediated anti-inflammatory effects.

It is now recognized that GPCR-targeting ligands can differentially modulate the activity of the various signaling pathways engaged by a single receptor with potential therapeutic advantage^39^. To capitalize on this phenomenon, known as functional selectivity or ligand-biased signaling, it is necessary to determine which pathways are essential for the therapeutic activity. In the present study, we used BRET^2^- and ebBRET-based biosensors to characterize the signaling repertoire of PAR2 in an attempt to identify the pathways responsible for the anti-inflammatory action of PAR2 blockade. The direct monitoring of G-protein activation confirmed the coupling promiscuity of PAR2^1,2,23–25,40^ engaging all G-protein sub-families, except for G_s_, in response to hTrypsin and SLIGKV-NH_2_. Previous works has suggested that the G_s_/cAMP production pathway could also be activated by PAR2^6,40,41^. We also found a weak cAMP production in response to trypsin but not SLIGKV-NH_2_ and the response to trypsin was also observed in PAR2 KO cells (Supplementary Fig. 12), indicating that the response may not be PAR2 specific.

Our data clearly reveal that I-287 acts as a biased NAM. Although a number of biased orthosteric ligands have now been described, I-287 adds to a short list of biased allosteric ligands (e.g., see refs. ^42–44^). It should also be noted that while most examples of biased ligands describe selectivity between G-protein activation and β-arrestins recruitment, I-287 not only displays bias between G proteins and β-arrestin but also among different G-protein subtypes, acting as a NAM for G_q_ and G_12/13_ but not G_i/o_. To date, only a limited number of antagonists targeting PAR2 have been described^45,46^. Among them, only one has been described as a biased antagonist until now. Indeed, GB88 selectively inhibits Ca^2+^ and PKC signaling whereas acting as a PAR2-agonist for the G_i/o_ pathway by reducing forskolin-stimulated cAMP, and in promoting ERK1/2 phosphorylation, RhoA activation, and βarrestin2 recruitment^7,9,47,48^. In contrast to GB88, I-287 displays no intrinsic agonist or inverse agonist activity towards any of the pathways tested (data not shown). Another PAR2 antagonist, I-191, was reported to be an antagonist blocking all the pathways tested, including Ca^2+^ release, ERK1/2 phosphorylation, RhoA activation, and inhibition of forskolin-induced cAMP accumulation^49^. Finally, four other compounds, the P2pal-18S i3 loop pepducin, the I-343, and AstraZeneca’s compounds AZ8838 and AZ3451, have been tested only for a limited subset of signaling pathways and found to be antagonists for all those tested^41,46,50^.

To our knowledge, our study is the first to report the ability of a PAR2 ligand to potently inhibit G_12/13_ activation. A previous study had reported the inhibition of PAR2-mediated RhoA activation by I-191^49^ but, given that both G_12/13_ and G_q_ can activate RhoA^51,52^, whether this inhibition was G_q_- or G_12/13_-mediated was unknown. Our study indicates that, in fact, both pathways can contribute. This is illustrated by the ability of the G_q_ inhibitor, YM-254890, to block PAR2-promoted SRF-RE induction only in G_12/13_ KO cells. Such complementary action of G_12/13_ and G_q_ in the activation of the RhoA pathways has previously been reported for the angiotensin receptor^29^. The inhibitory action of I-287 on G_q_ and G_12/13_ was further confirmed by its action on DAG and Ca^2+^ production, PKC and SRF-RE activation, as well as ERK and FAK phosphorylation.

Recently, several studies have reported different structures of the PAR2 receptor in complex with ligands, using crystallography or computational modeling studies^46,53,54^. Among them, the binding sites of two different NAMs (AZ8838 and AZ3451) were reported. Whereas AZ8838 binds in a pocket lined by residues from TM1–3, TM7, and ECL2, AZ3451 acts as a NAM by occupying a site formed by TM helices 2, 3, and 4, and faces the lipid bilayer^46^. Given the structure of I-287, it is unlikely that it could occupy these newly described binding pocket. For instance, attempts to overlay I-287 with AZ8838 and AZ3451 show no similarity between the molecules. More importantly, they do not share critical structural pharmacophore features that could support a common structure–activity relationships (SAR). Moreover, AZ8838 is completely buried in a small binding pocket that would be too small to accommodate I-287. Also, the SAR of I-287^22^ illustrates that large substituents can be appended to the piperazine portion of the molecule with no impact on potency, inconsistent with the enclosed small pocket for AZ8838. Concerning AZ3451, the planar shape of I-287 and its biophysical properties (namely its carboxylic acid) makes it unlikely that it could bind in the described highly lipophilic pocket. The identification of I-287 binding remains to be determined and will require additional studies.

One of the salient finding of our study is the fact that inhibition of G_q_ and G_12/13_ without affecting G_i/o_ activation or βarrestin recruitment is sufficient for the anti-inflammatory action of I-287. This is consistent with previous studies showing that Il-8 secretion in airway epithelial cells is regulated by PAR2-mediated activation of PLCβ/Ca^2+^ pathway^55^, and that neutrophil adhesion to lung A549 cancer cells is enhanced via the reorganization of actin and cytoskeleton through Rho/ROCK- and FAK-dependent pathways upon PAR2 stimulation^56^. Our study is the first to evaluate the impact of PAR2 antagonist on βarrestin recruitment and receptor internalization. Our results reveal that I-287 have no impact on these processes. PAR2-recruitement of β-arrestin has been shown to contribute to immune and cancer cells migration as scaffolding protein^57,58^ or to the mediation of pro-inflammatory effects in the airway^59^ and, thus, blocking βarrestin could present some advantages. However, given that blocking βarrestin and the resulting endocytosis would favor the maintenance of active receptor at the plasma membrane which could promote inflammatory response, a compound such as I-287 that does not favor or inhibit the recruitment of βarrestin could also represent an advantage. Consistent with the notion that blocking G_q_ and G_12/13_ but not βarrestin could be beneficial is the proposed role of β-arrestin recruitment by PAR2 in wound healing, notably through the activation of ERK1/2 patshway^60,61^. Whether the functional selectivity towards the G_q_ and G_12/13_ vs. G_i/o_ would represent a therapeutic advantage remains to be investigated. Indeed, although PAR2-mediated activation of G_i/o_ has been involved in breast cancer cell chemokinesis^62^ and lung adenocarcinoma cell lines migration^63^, this pathway has also been involved in expression induction of cyclooxygenase-2^64,65^, which displays protective functions in the gastrointestinal tract^66^.

**Fig. 8:**
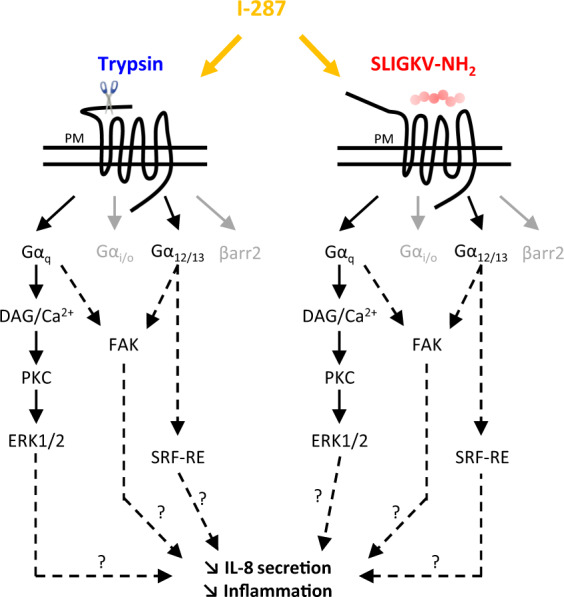
Effect of I-287 on intracellular signaling pathways induced by the two human PAR2 agonists, Trypsin, and SLIGKV-NH_2_. The pathways inhibited by I-287 are in black, whereas the unaffected pathways are in gray.

In conclusion, our study characterized I-287 as a new potent and functionally selective NAM for PAR2 that displays anti-inflammatory properties in vitro and in vivo. It also identified a subset of pathways which blockade is sufficient to promote PAR2-mediated anti-inflammatory effects (Fig. 8). The pathway-specific action of I-287 demonstrates that the development of functionally selective allosteric modulators with the desired physiological outcome is possible. This opens the path for the development of drugs selectively targeting only the therapeutically relevant pathways, thus reducing the liabilities associated with the blocking of the other pathways. Given that recent epidemiologic studies estimates that 5–7% of the population in western societies will be affected by one of IMIDs conditions and that a steady increase in this incidence is predicted^67,68^, the identification and the development of potent and selective inhibitors of PAR2 signaling pathways such as I-287 could therefore represent a promising therapeutics for the treatment of inflammation and nociception caused by inflammation, cancer, or injury.

## Methods

### Reagents

hTrypsin was from Sigma-Aldrich, SLIGKV-NH_2_ peptide was from Tocris, and SLIGRL-NH_2_ peptide from Abcam. Experimental protocols describing the synthesis of I-287 compound is provided in the Supplementary Methods.

### Plasmids

hPAR2 cDNA plasmid was purchased from Origen (IBD90.1 clone; catalog number SC322345), where the serine in position 291 has been mutated in threonine (S291T) to reproduce the hPAR2 phenotype observed in HEK293 and HCT 116 cells. For hPAR2-RlucII, we cloned hPAR2 sequence between the NheI and BamHI sites of pCDNA3.1/Zeo(+)-RlucII vector. Mouse PAR2 (mPAR2) cDNA plasmid was purchased from R&D Systems (catalog number RDC0167) and cloned between the BamHI and XbaI sites of pCDNA3.1/Zeo(+) vector. Human 3xHA-M3-mAChR has been purchased from UMR cDNA Resource Center (catalog number MAR030TN00). Plasmids encoding all the different non-tagged human G proteins used in this study were purchased from the Missouri University of Science and Technology (www.cdna.org). Non-tagged mouse G proteins were either PCR-amplified using a Riken mouse cDNA book^69^ or synthesized (GeneART, ThermoFisher). All the G proteins were subcloned into pCDNA3.1(+) vector. mGα_q_-RlucII were constructed by PCR-amplifying RlucII coding sequence and inserting it into pcDNA3.1(+) plasmid expressing the mGα_q_ using Gibson assembly. mGγ_1_-GFP10 was generated by subcloning the GFP10-tag sequence into pcDNA3.1-mGγ_1_. The constructs encoding the G_12/13_ binding domain of the human p115-RhoGEF (residues 1–244) tagged with RlucII were done by PCR amplification from IMAGE clones (OpenBiosystems) and subcloned by Gibson assembly in pCDNA3.1 Hygro(+) GFP10-RlucII, replacing GFP10. A peptidic linker (RLKLPAT) is present between RlucII and the G_12/13_ binding domain. The following plasmids were previously described: human G-protein subunits, GRK2-GFP10, and Gγ5-RlucII biosensors^26^; Gα_q_-118-RlucII, Gα_i2_-91-RlucII, Gα_oA_-99-RlucII, Gα_12_-136-RlucII, Gα_13_-130-RlucII, and GFP10-Gγ_1_ or -Gγ_2_^26–28^; mGα_i2_-RlucII and mGγ_2_-GFP10 mouse biosensors^70^; unimolecular BRET^2^-based DAG, PKC, and EPAC biosensors^29,32^, and rGFP-CAAX with human or mouse βarrestin2–RlucII^34,70^ and HA-TPαR^71^.

### Cell culture

All cell culture reagents were from Wisent, Inc. HEK293, mouse rectal carcinoma (CMT-93), human colon carcinoma (HCT 116), and human lung carcinoma (A549) cells were obtained from the American Type Culture Collection. All cell lines were cultured in complete medium containing Dulbecco’s modified Eagle’s medium supplemented with 10% FBS and 1% antibiotics (100 U/mL penicillin and 100 μg/mL streptomycin; PS), except for HCT 116 cell line, which was grown in McCoy’s 5a Medium Modified supplemented with 10% FBS and 1% PS. Cells were passaged weekly and incubated at 37 °C in an humidified atmosphere with 5% CO_2_. HEK293 cells devoid of functional Gα_12_ and Gα_13_ proteins (ΔG_12_/G_13_), a gift from A. Inoue (Tohoku University, Sendai, Miyagi, Japan), were previously described^29,31^. HEK293 cells devoid of functional βarrestin1/2 by the CRISPR/Cas9 system (Δβarrestin1/2), a gift from S. Laporte (McGill University, Montreal, Quebec, Canada), were previously described^72^. HEK293 cells devoid of functional hPAR2 have been generated by the CRISPR/Cas9 system.

### Bioluminescence resonance energy transfer measurement

Forty-eight hours before the experiments, 1 μg of total DNA (adjusted with salmon sperm DNA; Invitrogen) was used to transfect 3.5 × 10^5^ cells per mL using linear polyethylenimine (PEI, 1 mg/mL; Polysciences) diluted in NaCl (150 mM pH 7.0) as a transfecting agent (3 : 1 PEI/DNA ratio). Cells were immediately seeded (3.5 × 10^4^ cells/well) in poly-ornithine- (Sigma-Aldrich) coated 96-well white microplates (PerkinElmer). Cells were maintained in culture for the next 48 h and BRET experiments carried out.

G-protein activation profile was elucidated with the GRK-based BRET^2^ biosensor, based on the competition between Gα subunit and GRK2 protein to bind the βγ dimer, by transfecting cells with hPAR2 and human Gβ_1_, RlucII-Gγ_5_, and GRK2-GFP10 along with the indicated human Gα subunit. hPAR2-mediated activation of Gα proteins was confirmed employing human BRET^2^ biosensors based on the separation of human Gα-RlucII and GFP10-Gγ in the presence of Gβ_1_^26–28^. For mPAR2, cells were co-transfected with the mouse biosensors mGα_q_-RlucII/mGγ_1_-GFP10 or mGα_i2_-RlucII/mGγ_2_-GFP10^70^. DAG production and PKC activation were evaluated in cells transiently expressing hPAR2 or mPAR2 and unimolecular BRET^2^-based DAG or PKC biosensors^29^. β-Arrestin recruitment to the plasma membrane was determined using βarrestin2–RlucII and a plasma membrane marker for the use in ebBRET assays, composed of the *Renilla reniformis* GFP (rGFP) fused at the C terminus to a plasma membrane-targeting domain composed of the polybasic sequence and prenylation CAAX box of KRas (rGFP-CAAX)^34^. PAR2 internalization was evaluated by measuring the disappearance of hPAR2-RlucII from the plasma membrane labeled with rGFP-CAAX. The effector membrane translocation BRET biosensors p115-RhoGEF-RlucII has been used to monitor activation of Gα_12/13_ proteins by following the recruitment of this selective effector of activated Gα_12/13_ subunits to the plasma membrane tagged with the anchored rGFP-CAAX. A summary of BRET-based biosensors used in the study is presented in Supplementary Table 1.

The day of the experiment, cells were washed with phosphate-buffered saline (PBS) and incubated in Tyrode Hepes buffer (137 mM NaCl, 0.9 mM KCl, 1 mM MgCl_2_, 11.9 mM NaHCO_3_, 3.6 mM NaH_2_PO_4_, 25 mM HEPES, 5.5 mM d-Glucose, and 1 mM CaCl_2_ pH 7.4) for 1 h at 37 °C. Cells were then treated with increasing concentrations of ligands for the indicated times at 37 °C. The luciferase substrates Coelenterazine 400a (2.5 µM, NanoLight Technologies) or Prolume purple for internalization experiments (2 µM, NanoLight Technologies) were added to the wells 5 min before measurements. Plates were read on the TriStar² LB 942 Multimode Microplate Reader (Berthold Technologies) with the energy donor filter (410 ± 80 nm; RlucII) and energy acceptor filter (515 ± 40 nm; GFP10 and rGFP-CAAX). BRET signal (BRET^2^) was determined by calculating the ratio of the light intensity emitted by the acceptor (515 nm) over the light intensity emitted by the donor (410 nm). The agonist-promoted BRET signal (ΔBRET) refers to the difference in BRET recorded from cells treated with agonist and cells treated with vehicle. For the agonist dose–response curves, the percentage of the response of the indicated condition (% *E*_max_ of agonist or transfection condition) was calculated from the ΔBRET value obtained from a given condition divided by the ΔBRET obtained from the control condition (e.g., hTrypsin for hPAR2, SLIGRL-NH_2_ for mPAR2, or control cells) and multiplied by 100. Data are expressed as mean of at least three independent experiments ± SEM.

To evaluate the impact of compound I-287 on PAR2 signaling, cells were preincubated for 15–30 min with dimethyl sulfoxide (DMSO) or I-287 at the indicated concentrations, always keeping DMSO at 1% final concentration in each well. Cells were then treated with vehicle or an EC_80_ concentration of the indicated agonist, determined from the dose–response curves obtained in PAR2 signaling characterization, and BRET signal was recorded as described above.

### Ca^2+^ measurement

Forty-eight hours after seeding (3.5 × 10^4^ cells/well) in poly-ornithine-coated, 96-well clear-bottom black microplates (Perkin Elmer), cells were incubated with 100 μL of a Ca^2+^-sensitive dye-loading buffer (FLIPR calcium 5 assay kit, Molecular Devices) containing 2.5 mM probenecid for 1 h at 37 °C in a 5% CO_2_ incubator. During a data run, cells in individual wells were exposed to various concentrations of drugs and fluorescent signals were recorded every 1.5 s for 3 min using the FlexStation II microplate reader (Molecular Devices). Increases in intracellular Ca^2+^ levels were determined by subtracting basal values from peak values. To assess the role of G proteins in PAR2-mediated Ca^2+^ release, cells were pretreated either with PTX (100 ng/mL, 18 h; List Biological Laboratories) or YM-254890 (1 µM, 30 min; Wako Pure Chemical Industries) before agonist stimulation.

### cAMP assay

Cells were transiently transfected with hPAR2 and EPAC BRET²-based biosensor using PEI and immediately seeded (3.5 × 10^4^ cells/well) in poly-ornithine-coated, 96-well white microplates. Forty-eight hours later, cells were washed with PBS and incubated in Tyrode Hepes buffer for 1 h at 37 °C. To measure cAMP modulation in response to G_i/o_-activation, cells were first treated for 5 min with increasing concentrations of SLIGKV-NH_2_ and then stimulated with forskolin (1.5 µM, 5 min) in the presence of Coelenterazine 400a (2.5 µL) and BRET signal was recorded as described above.

### SRF-RE reporter gene assay

Cells were transiently transfected with hPAR2 and the pGL4.34[luc2P/SRF-RE/Hygro] vector (Promega) that contains a SRF-RE driving the transcription of a firefly luciferase reporter gene (luc2P) upon SRF activation. The pCDNA3.1(+)-RlucII plasmid expressing *Renilla* luciferase reporter gene was systematically used as an internal control to normalize for transfection efficiency. Cells were immediately seeded (3.5 × 10^4^ cells/well) after transfection in 96-well white microplates (Perkin Elmer). Five hours after, medium was changed for respective culture medium supplemented with 0.5% FBS and cells were incubated for 16 h at 37 °C. Impact of compound I-287 on PAR2-induced SRF-RE activation was tested by preincubating cells for 30 min with DMSO or I-287 (10 µM) diluted in culture medium containing 0.5% FBS, and stimulating cells with hPAR2 agonists (10 U/mL hTrypsin or 100 µM SLIGKV-NH_2_) during 6 h at 37 °C. Firefly and *Renilla* luciferase activities were measured using the Dual-Glo® Luciferase Assay System (Promega) according to the manufacturer’s instructions.

### Western blot analysis

Cells were transfected with hPAR2 using PEI and seeded in six-well plates (10^6^ cells/well) in complete medium. Twenty-four hours later, cells were starved overnight in serum-free medium. The day after, cells were preincubated with DMSO or I-287 (10 µM, 30 min), or with PTX (100 ng/mL, 18 h), YM-254890 or Gö 6983 (1 µM, 30 min; Calbiochem), or CT04 (1 µg/mL, 6 h; Cytoskeleton, Inc.), and treated with 1 U/mL hTrypsin or 100 µM SLIGKV-NH_2_ for the indicated time. Cells were then washed with ice-cold PBS and lysed in a buffer containing 10 mM Tris buffer (pH 7.4), 100 mM NaCl, 1 mM EDTA, 1 mM EGTA, 10% SDS, 1% Triton X-100, 10% Glycerol supplemented with protease and phosphatase inhibitors cocktails (Roche). Cell lysates were centrifuged at 20,000 × *g* for 30 min at 4 °C. Equal amounts of proteins were separated by SDS-PAGE and transferred onto polyvinylidene fluoride membrane. Proteins were detected using specific antibodies targeting the protein of interest: phospho-FAK (Y397; catalog number: ab81298, 1 : 1000 dilution; Abcam), total-FAK (catalog number: ab40794, 1 : 1000 dilution; Abcam), phospho-ERK1/2 (catalog number 9101; 1 : 1000 dilution; Cell Signaling), and total ERK1/2 (catalog number 9102; 1 : 1000 dilution; Cell Signaling). Western blottings were visualized using enhanced chemiluminescence and detection was performed using a ChemiDoc MP Imaging System (BioRad). Relative densitometry analysis on protein bands was performed using MultiGauge software (Fujifilm). Results were normalized against control bands. Uncropped immunoblots are shown in Supplementary Fig. 13.

### Investigation of IL-8 release

HCT 116 or A549 cells were seeded (2.5–3 × 10^5^ cells/well) and grown for 24 h, before transfection with hPAR2 using the X-tremeGENE™ HP DNA Transfection Reagent (Roche) according to the manufacturer’s instructions. Twenty-four hours after, cells were starved overnight before pretreatment with DMSO or I-287 (10 µM, 30 min), followed by incubation with hTrypsin (1 U/mL) or SLIGKV-NH_2_ (100 µM) for 6 h. TNF-α (10 ng/mL) for 6 h was used as a positive control. Culture medium was collected and the amount of IL-8 secreted into the supernatants was quantified by the DuoSet ELISA human CXCL8/IL-8 immunoassay kit (R&D Systems).

### Animals

Adult male C57BL/6J mice weighing 25–30 g (Charles River Laboratories, Canada) were maintained on a 12 h light/dark cycle in groups of three to four per cage. After arrival, mice were acclimatized for a week before any experimental procedure. Water and food were available ad libitum. Maximum efforts have been made to limit the number of animals used and their suffering. All procedures were performed in accordance with the Canadian Council on Animal Care and with the International Association for the Study of Pain guidelines for pain research on animals. Procedures were also approved by the local Animal Care Committee at the Université de Sherbrooke and were part of protocol 242–14.

### CFA-induced paw inflammation

To evaluate the in vivo anti-inflammatory activity of I-287 compound, we used the mouse CFA-induced paw edema model. Briefly, inflammation was induced by the intraplantar injection of 50 µL of CFA (Calbiochem) to the left hindpaw of mice. CFA was prepared as a 1 : 1 emulsion of paraffin oil and 0.9% saline solution, complemented by the addition of lyophilized bacterial membranes to reach a concentration of 100 µg/50 µL (*Mycobacterium butyricum*, BD Difco, Fisher Scientific) as previously described^73^. Before CFA injection, mice were weighed and the volume of their ipsilateral hindpaws was measured using a plethysmometer (IITC Life Science, Inc.). Each hindpaw was soaked to the medial malleolus and the paw volume determined in microliters. One hour after CFA injection, mice were treated either by vehicle (5% Methylpyrrolidone (Sigma-Aldrich) and 95% of 10% d-α-Tocopherol polyethylene glycol 1000 succinate (TPGS, Sigma-Aldrich)), I-287 (50 mg/kg), or Ibuprofen (140 mg/kg; Lot #108K1067; Sigma-Aldrich), as a positive control of anti-inflammatory activity. Drugs were administered by gavage in a volume of 500 µL/30 g of body weight. The dose of Ibuprofen was chosen in accordance to^74^. The paw volume was then evaluated each hour during a 5 h period following CFA injection by an experimenter blind to the treatment.

### Statistics and reproducibility

Results are expressed as mean ± SEM and the number of independent experiments is indicated in the legend of each figure. Data were analyzed in GraphPad Prism 8 Software using Student’s *t*-test and analysis of variance followed by Dunnett’s or Tuckey’s post hoc tests for multiple comparisons, performed as appropriate (see figure legends). Significance was determined as *p* < 0.05. Concentration–response curves were fitted in GraphPad Prism using a three-parameter fitting with a standard Hill slope of 1 (agonist) or −1 (inhibitor).

### Reporting summary

Further information on research design is available in the Nature Research Reporting Summary linked to this article.

## Supplementary information

Supplementary Information

Reporting Summary

## Data Availability

All data that support the findings of this study are available from the corresponding author upon request. Source data for the graphs and charts in the main figures are available in Supplementary Data 1.
