# Supplementary material for: The PAR2 inhibitor I-287 selectively targets Gαq and Gα12/13 signaling and has anti-inflammatory effects
Source: Commun Biol. 2020 Nov 27;3:719. doi: 10.1038/s42003-020-01453-8 (PMC7695697; doi:10.1038/s42003-020-01453-8)
Supplement: Supplementary file 1 — Supplementary Information [file 42003_2020_1453_MOESM1_ESM.pdf]

Supplementary Information from:

The PAR2 inhibitor I-287 selectively targets Gα_q and G$\alpha_{12/13}$ signaling and has anti-inflammatory effects

Charlotte Avet[1], Claudio Sturino[2,4], Sébastien Grastilleur[3], Christian Le Gouill[1], Meriem Semache[1,5], Florence Gross[1,5], Louis Gendron[3], Youssef Bennani[2,6], Joseph A. Mancini[2,7], Camil E. Sayegh[2,8] and Michel Bouvier[1]

[1]Institute for Research in Immunology and Cancer, and Department of Biochemistry and Molecular Medicine, Université de Montréal, Montréal, QC H3C 1J4, Canada;

[2]Vertex Pharmaceuticals (Canada) Inc.; Laval, Québec H7V 4A7, Canada.

[3]Département de Pharmacologie-Physiologie, Université de Sherbrooke, Centre de Recherche du CHU de Sherbrooke, Centre d'Excellence en Neurosciences de l'Université de Sherbrooke, and Institut de Pharmacologie de Sherbrooke, Sherbrooke, QC J1H 5N4, Canada.

[4]Current address: Paraza Pharma Inc.; Saint-Laurent, QC H4S 2E1, Canada.

[5]Current address: Domain Therapeutics North America; Saint-Laurent, Québec, H4S 1Z9, Canada.

[6]Current address: AdMare BioInnovations; Saint-Laurent, Québec, H4S 1Z9, Canada.

[7]Current address: Vertex Pharmaceuticals Inc.; Boston, MA 02210, United States.

[8]Current address: Ra Pharmaceuticals, Inc.; Cambridge, MA 02140, United States.

Corresponding Author: Michel Bouvier, IRIC - Université de Montréal, P.O. Box 6128 Succursale Centre-Ville, Montréal, Qc. Canada, H3C 3J7. Tel: +1-514-343-6319. Fax: +1-514-343-6843. Email: michel.bouvier@umontreal.ca

Supplementary Tables

Supplementary Table 1: Summarize of BRET-based biosensors used in the study

Target	Donor	Acceptor	Reference
Gα protein activation<br>GRK2 sensor	RlucII-Gγ_5	GRK2-GFP10	Mende *et al.* 2018
Gα and Gγ subunits separation	Gα-RlucII	GFP10-Gγ	Gales *et al.* 2006;<br>Quoyer *et al.* 2013;<br>Mende *et al.* 2018;<br>Ehrlich *et al.* 2019
DAG production	GFP10-linker-RlucII-C1b		Namkung *et al.* 2018
PKC activation	GFP10-FHA1/2-linker-pPKC1/2-RlucII		Namkung *et al.* 2018
cAMP production	GFP10-EPAC1-RlucII		Leduc *et al.* 2009
βarrestin2 recruitment to PM	βarrestin2–RlucII	rGFP-CAAX	Namkung *et al.* 2016
PAR2 disappearance from PM	hPAR2-RlucII	rGFP-CAAX	This study
Selective G$\alpha_{12/13}$ activation	p115-RhoGEF-RlucII	rGFP-CAAX	This study

PM : plasma membrane

Supplementary Figures

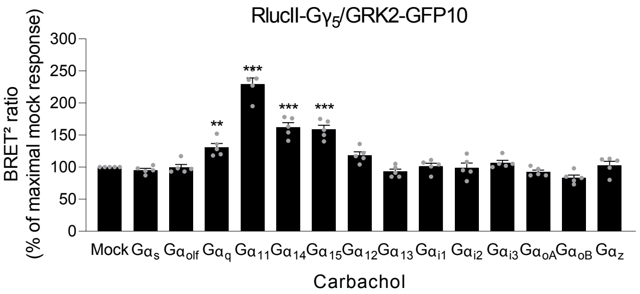

Supplementary Fig. 1: G proteins activation profile of M3-mAChR in response to carbachol in HEK293 cells. G proteins activation profile induced by carbachol (100 µM, 15 min) in HEK293 cells expressing M3-mAChR and components of the GRK2-based G protein activation sensor (RlucII-Gγ_5, GRK2-GFP10, Gβ_1 and the indicated Gα). Results are expressed as BRET² ratio in % of maximal response obtained in mock condition (mean ± SEM; n = 5; One-way ANOVA followed by Dunnett's post hoc: **$p < 0.01$ and ***$p < 0.001$ compared to mock condition).

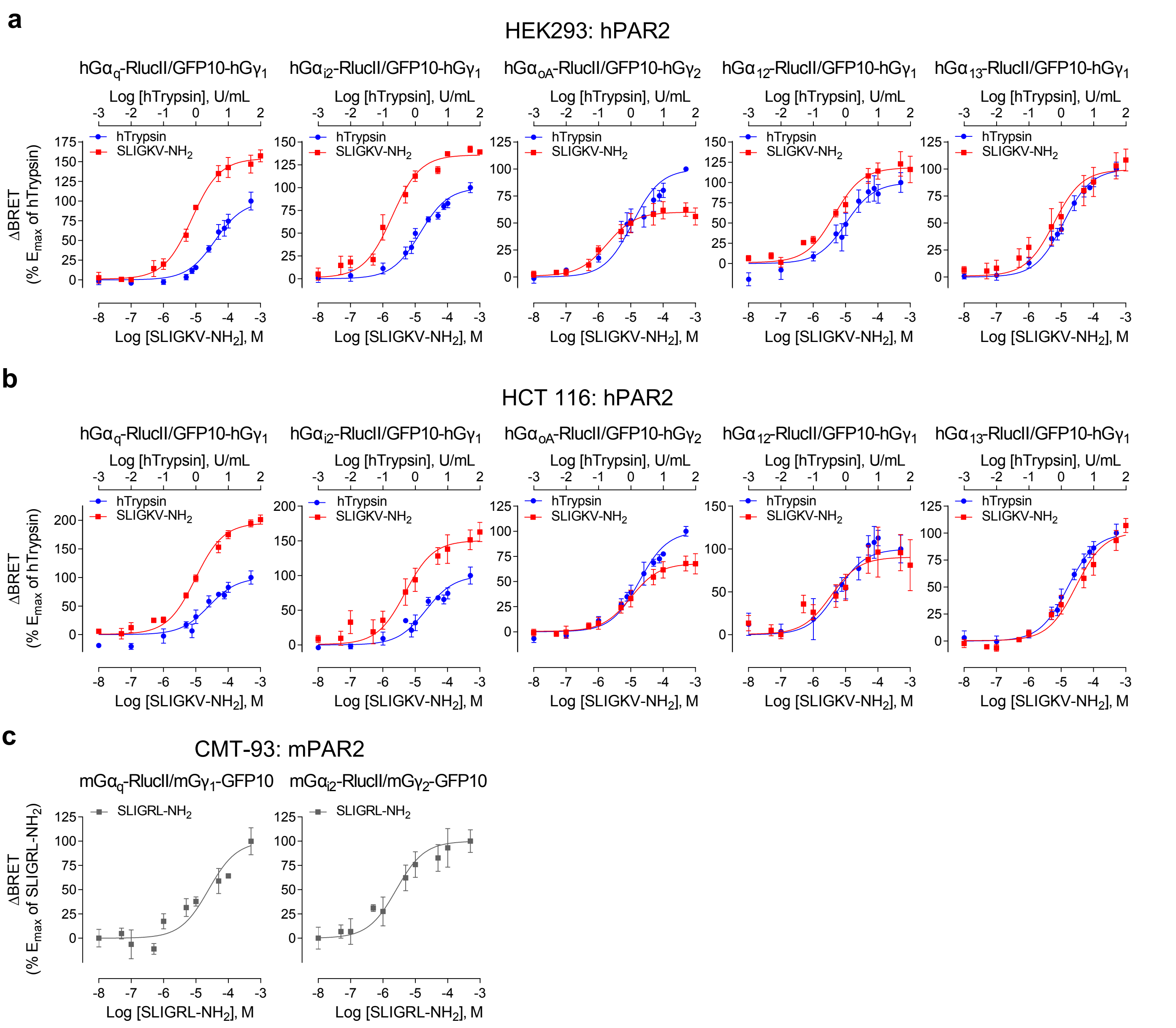

d

Pathway	Agonist	HEK293: hPAR2 pEC50	HEK293: hPAR2 Emax (% hTrypsin)	HCT 116: hPAR2 pEC50	HCT 116: hPAR2 Emax (% hTrypsin)	CMT-93: mPAR2 pEC50	CMT-93: mPAR2 Emax (% SLIGRL-NH2)
$G\alpha_q$	hTrypsin (U/mL)	0.64 ± 0.08	100	0.40 ± 0.08	100	-	-
	SLIGKV-NH2 (M)	-5.12 ± 0.06	153.2 ± 4.0	-5.01 ± 0.05	195.3 ± 4.3	-	-
	SLIGRL-NH2 (M)	-	-	-	-	-4.61 ± 0.11	100
$G\alpha_{i2}$	hTrypsin (U/mL)	0.15 ± 0.04	100	0.33 ± 0.06	100	-	-
	SLIGKV-NH2 (M)	-5.71 ± 0.07	135.1 ± 3.7	-5.31 ± 0.12	150.1 ± 7.7	-	-
	SLIGRL-NH2 (M)	-	-	-	-	-5.59 ± 0.12	100
$G\alpha_{oA}$	hTrypsin (U/mL)	0.05 ± 0.07	100	0.26 ± 0.04	100	-	-
	SLIGKV-NH2 (M)	-5.73 ± 0.12	59.3 ± 2.7	-4.99 ± 0.11	67.6 ± 3.4	-	-
$G\alpha_{12}$	hTrypsin (U/mL)	0.01 ± 0.09	100	-0.27 ± 0.11	100	-	-
	SLIGKV-NH2 (M)	-5.35 ± 0.09	117.5 ± 4.5	-5.45 ± 0.22	90.0 ± 8.1	-	-
$G\alpha_{13}$	hTrypsin (U/mL)	0.06 ± 0.04	100	0.20 ± 0.04	100	-	-
	SLIGKV-NH2 (M)	-5.22 ± 0.13	98.7 ± 5.7	-4.54 ± 0.09	100.7 ± 4.8	-	-

Supplementary Fig. 2: G proteins activation profiles of hPAR2 in HEK293 and HCT 116 cells, and mPAR2 in CMT-93 cells. a, b Dose-response curves of G proteins activation induced after 1 min stimulation with increasing concentrations of hTrypsin or SLIGKV-NH_2 in HEK293 (**a**) and HCT 116 (**b**) cells co-expressing hPAR2, $G\beta_1$ and the human BRET²-based α/βγ dissociation biosensors (hGα-RlucII and GFP10-$hG\gamma_1$ for $G\alpha_q$, $G\alpha_{i2}$, $G\alpha_{12}$ and $G\alpha_{13}$, or GFP10-$hG\gamma_2$ for $G\alpha_{oA}$). Data presented in **a** are the same as in **Fig. 1e-i**, but with results expressed as ΔBRET in % E_{max} of hTrypsin (mean ±SEM; n = 3). **c** Dose-response curves of G proteins activation induced by increasing concentrations of SLIGRL-NH_2 in CMT-93 cells co-expressing mPAR2, $G\beta_1$ and the mouse BRET²-based biosensors $mG\alpha_q$-RlucII/$mG\gamma_1$-GFP10 or $mG\alpha_{i2}$-RlucII/$mG\gamma_2$-GFP10. Results are expressed as ΔBRET in % E_{max} of SLIGRL-NH_2 (mean ± SEM; n = 4). **d** Summary of potencies (pEC_{50}) and relative efficacies (E_{max}) of ligand-induced Gα proteins activation by the human and murine PAR2. Data are the mean ± SEM deduced from the concentration-response curves above.

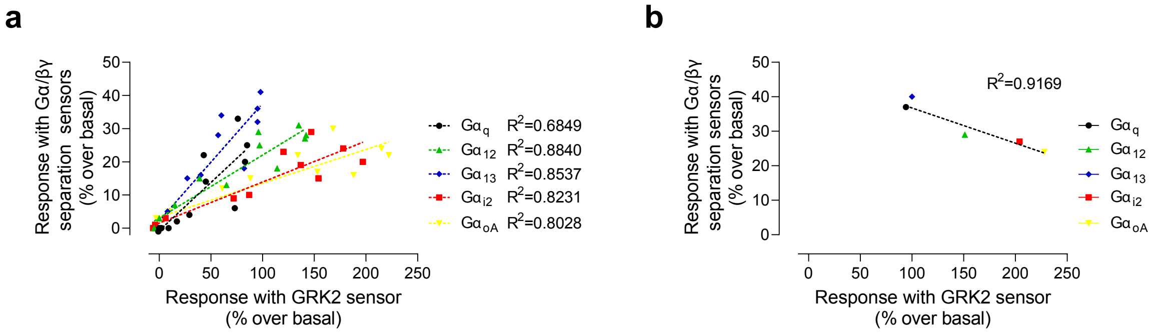

Supplementary Fig. 3: Bias plot for hTrypsin-mediated activation of G proteins by PAR2. a Responses obtained for each G protein using the sensor based on Gα and Gγ subunits separation (ordinates) were expressed as a function of the responses obtained using the G protein activation GRK2 sensor (abscissae) produced by common concentrations of hTrypsin. Results are expressed as $BRET^2$ ratio in % over respective basal response (mean ± SEM; n = 3-6). The resulting concentration-response curves were fitted in GraphPad Prism using a linear regression (dotted lines) and R^2 is indicated for each pathway. **b** Maximal responses obtained for each G protein using the Gα/Gβγ separation sensor (ordinates) in response to hTrypsin were expressed as a function of the maximal responses obtained using the G protein activation GRK2 sensor (abscissae). Results are expressed as $BRET^2$ ratio in % over respective basal response (mean ± SEM; n = 3-6). The different G protein points were fitted using a linear regression (dotted line) and R^2 is indicated.

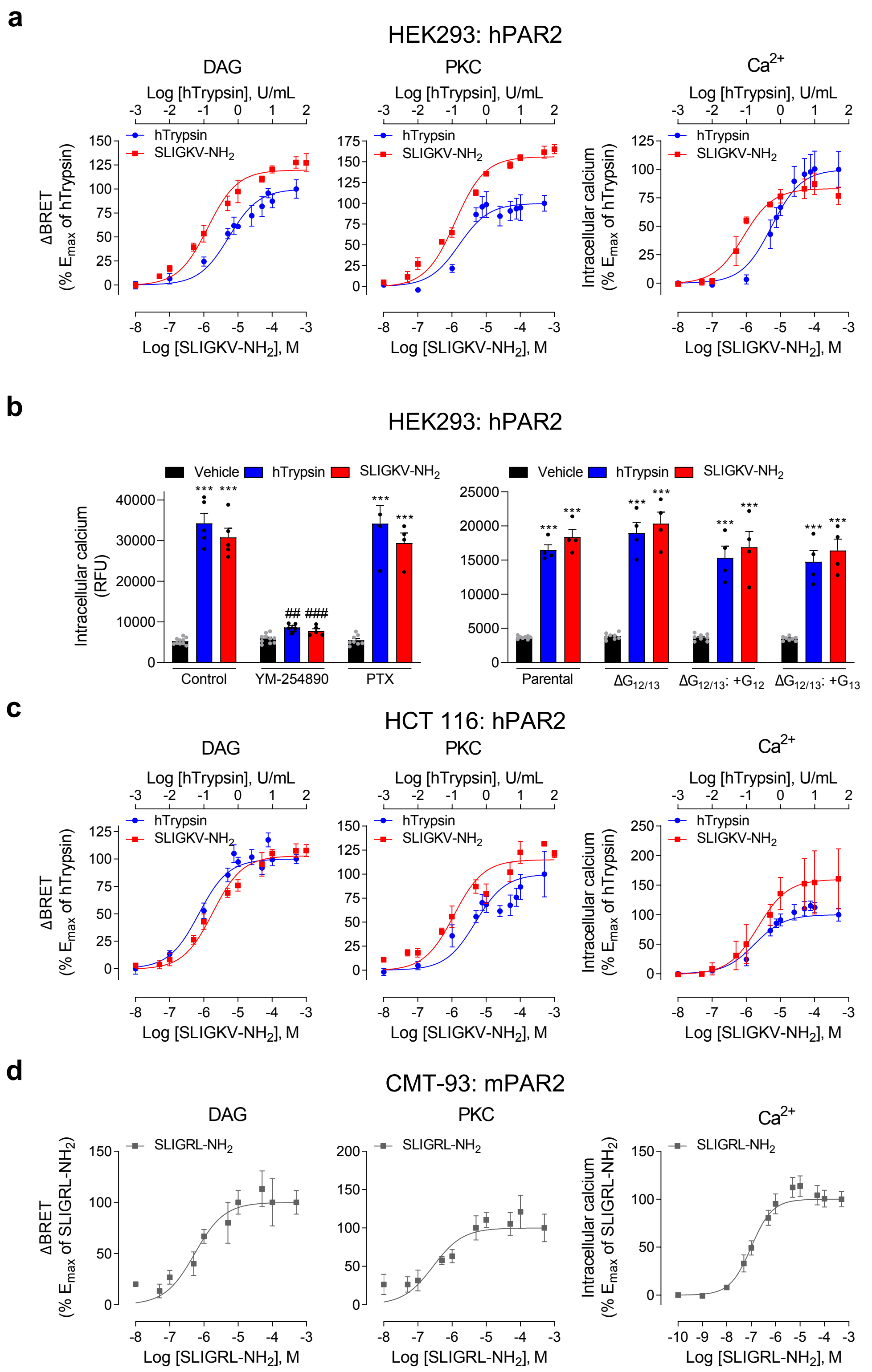

e

Pathway	Agonist	HEK293: hPAR2		HCT 116: hPAR2		CMT-93: mPAR2	
		pEC_{50}	E_{max} (% hTrypsin)	pEC_{50}	E_{max} (% hTrypsin)	pEC_{50}	E_{max} (% SLIGRL-NH_2)
DAG	hTrypsin (U/mL)	-0.27 ± 0.06	100	-1.15 ± 0.09	100	-	-
	SLIGKV-NH_2 (M)	-5.88 ± 0.07	120.4 ± 3.1	-5.74 ± 0.07	103.5 ± 2.8	-	-
	SLIGRL-NH_2 (M)	-	-	-	-	-6.27 ± 0.13	100
PKC	hTrypsin (U/mL)	-0.79 ± 0.12	100	-0.31 ± 0.11	100	-	-
	SLIGKV-NH_2 (M)	-5.90 ± 0.05	156.9 ± 3.0	-5.94 ± 0.10	115.7 ± 4.2	-	-
	SLIGRL-NH_2 (M)	-	-	-	-	-6.56 ± 0.16	100
Ca^{2+}	hTrypsin (U/mL)	-0.26 ± 0.10	100	-0.76 ± 0.11	100	-	-
	SLIGKV-NH_2 (M)	-6.08 ± 0.11	83.8 ± 3.5	-5.65 ± 0.25	161 ± 16.2	-	-
	SLIGRL-NH_2 (M)	-	-	-	-	-7.00 ± 0.09	100

Supplementary Fig. 4: Characterization of G proteins downstream signaling pathways activated by hPAR2 in HEK293 and HCT 116 cells and by mPAR2 in CMT-93 cells. a, c, d Dose-response curves of DAG production, PKC activation and Ca^{2+} release, induced by increasing concentrations of hTrypsin or human (SLIGKV-NH_2) and mouse (SLIGRL-NH_2) PAR2-activating peptides. DAG (*left panel*) and PKC (*central panel*) were measured in HEK293 (**a**), HCT 116 (**c**), or CMT-93 (**d**) cells expressing either hPAR2 or mPAR2, and the corresponding unimolecular BRET²-based biosensors. Data presented in **a** are the same as in **Fig. 2a-c**, but with results expressed as ΔBRET in % E_{max} of hTrypsin for hPAR22 or SLIGRL-NH_2 for mPAR2 (mean ±SEM; n = 3). Ca^{2+} release (*right panel*) was measured in HEK293 (**a**), HCT 116 (**c**), or CMT-93 (**d**) cells expressing endogenous hPAR2 or mPAR2, respectively. Results are expressed as RFU peak value in % E_{max} of hTrypsin or SLIGRL-NH_2 (mean ±SEM; n = 4-7). **b** *Left panel*; Ca^{2+} release induced by hTrypsin (10 U/mL) or SLIGKV-NH_2 (100 µM) in HEK293 cells pretreated with YM 254890 (1 µM, 30 min) or PTX (100 ng/mL, 18 h). *Right panel*; Ca^{2+} release induced by hTrypsin (10 U/mL) or SLIGKV-NH_2 (100 µM) in HEK293T wild type cells (parental), or in HEK293T $G\alpha_{12/13}$ knockout cells transfected with control DNA ($\Delta G_{12/13}$) or either $G\alpha_{12}$ or $G\alpha_{13}$ (mean ±SEM; n = 4-5; Two-way ANOVA followed by Tukey's post-hoc: $***p < 0.001$ compared to respective basal condition, $^{###}p < 0.001$ compared to control cells with the respective agonist). **e** Summary of potencies (pEC_{50}) and relative efficacies (E_{max}) of DAG and Ca^{2+} productions and PKC activation induced by PAR2 ligands. Data are the mean ±SEM deduced from the concentration-response curves above.

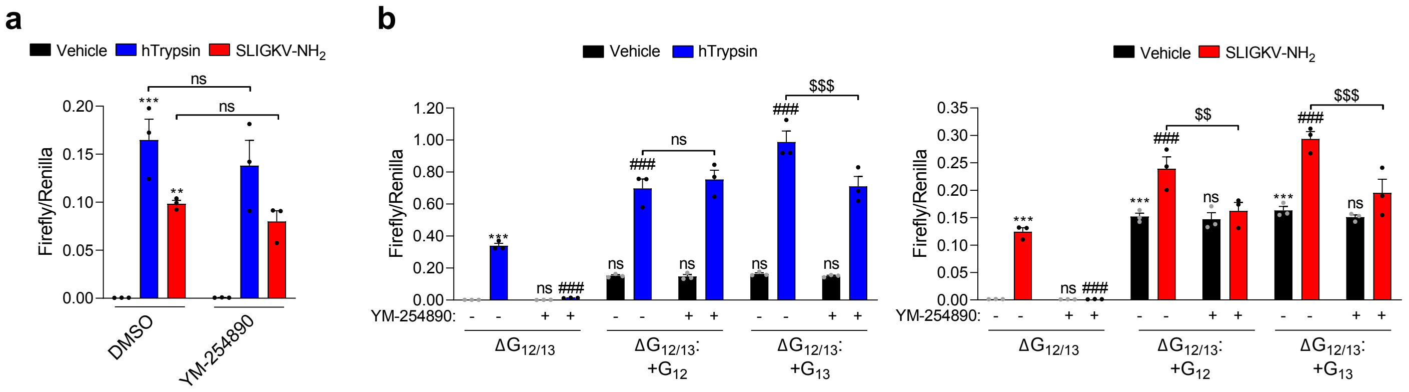

Supplementary Fig. 5: hPAR2 promoted SRF-RE reporter gene activation is mediated by $G_{12/13}$ but not $G_{q/11}$ proteins. a Impact of YM-254890 (1 µM, 30 min) on hPAR2-promoted SRF-RE reporter gene activation induced after 6 h stimulation with hTrypsin (10 U/mL) or SLIGKV-NH_2 (100 µM) in HEK293 cells expressing hPAR2. Results are expressed as % of the response induced by respective agonists in absence of I-287 (mean ± SEM; n = 3; Two-way ANOVA followed by Tukey's post-hoc: $^{**}p < 0.01$ and $^{***}p < 0.001$ compared to control cells). **b** Measurement of SRF-RE reporter gene activation induced by hTrypsin (10 U/mL) or SLIGKV-NH_2 (100 µM) in HEK293T $G\alpha_{12/13}$ knockout cells transfected with control DNA ($\Delta G_{12/13}$) or with $G\alpha_{12}$ or $G\alpha_{13}$, with or without pre-treatment with YM-254890 (1 µM, 30 min) (mean ±SEM; n = 3; Two-way ANOVA followed by Tukey's post-hoc: $^{***}p < 0.001$ compared to $\Delta G_{12/13}$ untreated cells, $^{\#\#\#}p < 0.001$ compared to $\Delta G_{12/13}$ agonist-treated cells and $^{\$\$}p < 0.01$, $^{\$\$\$}p < 0.001$ compared to equivalent cells minus YM-254890 pre-treatment, ns = non-significant).

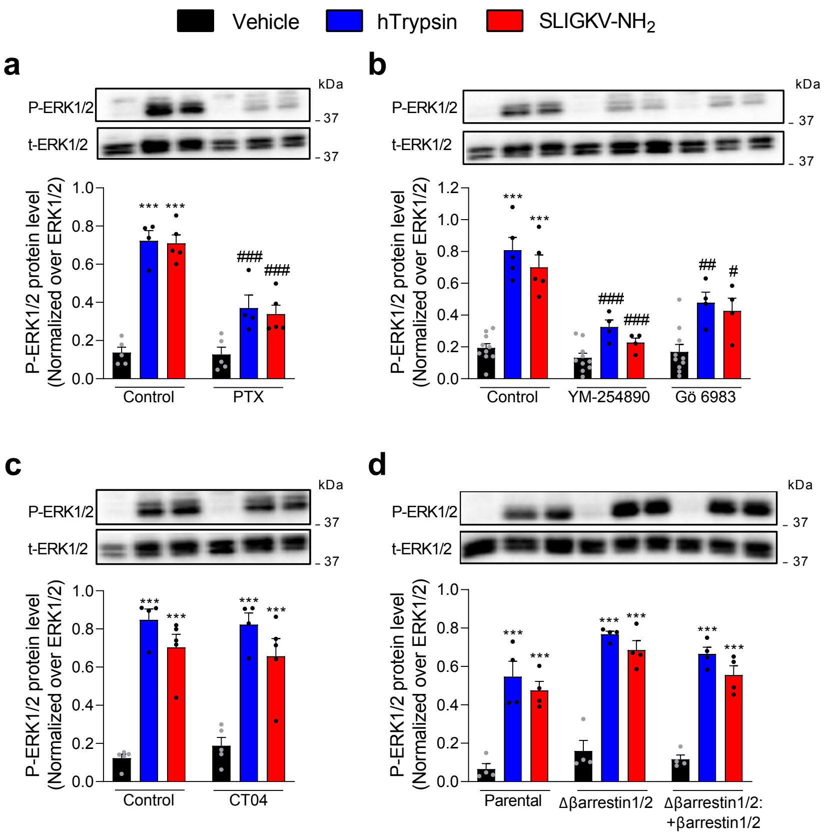

Supplementary Fig. 6: hPAR2-induced ERK1/2 phosphorylation is mediated by $G_{i/o}$ and G_q/PKC proteins. **a-c** Measurement of ERK1/2 phosphorylation induced by hTrypsin (1 U/mL) or SLIGKV-NH_2 (100 µM) for 10 min in hPAR2 expressing HEK293 cells pretreated with PTX (100 ng/mL, 18 h; **a**), YM 254890 or Gö 6983 (1 µM, 30 min; **b**) or CT04 (1 µg/mL, 6 h; **c**). **d** Measurement of ERK1/2 phosphorylation induced by hTrypsin (1 U/mL) or SLIGKV-NH_2 (100 µM) in HEK293 wild type ells (parental), or in HEK293 βarrestin1/2 knockout cells transfected with control DNA (Δβarrestin1/2) or with βarrestin1/2. Representative immunoblots of ERK1/2 phosphorylation are shown. Western blots were quantified and expressed as the ratio of phosphorylated ERK (P-ERK1/2) protein level normalized over total ERK (t-ERK1/2) protein (mean ± SEM; n = 4-5; Two-way ANOVA followed by Tukey's post-hoc: ***p < 0.001 compared to control or parental cells with vehicle; $^{\#}p < 0.05$, $^{\#\#}p < 0.01$ and $^{\#\#\#}p < 0.001$ compared to control cells with respective agonist).

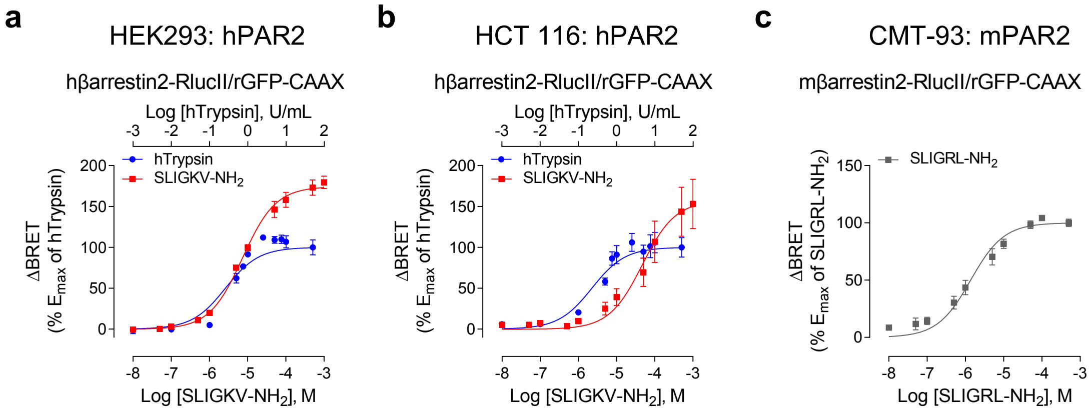

d

Pathway	Agonist	HEK293: hPAR2 pEC50	HEK293: hPAR2 Emax (% hTrypsin)	HCT 116: hPAR2 pEC50	HCT 116: hPAR2 Emax (% hTrypsin)	CMT-93: mPAR2 pEC50	CMT-93: mPAR2 Emax (% SLIGRL-NH2)
βarrestin2	hTrypsin (U/mL)	-0.58 ± 0.08	100	-0.65 ± 0.09	100	-	-
	SLIGKV-NH2 (M)	-5.13 ± 0.04	174.5 ± 3.0	-4.33 ± 0.14	157.3 ± 12.1	-	-
	SLIGRL-NH2 (M)	-	-	-	-	-5.86 ± 0.05	100

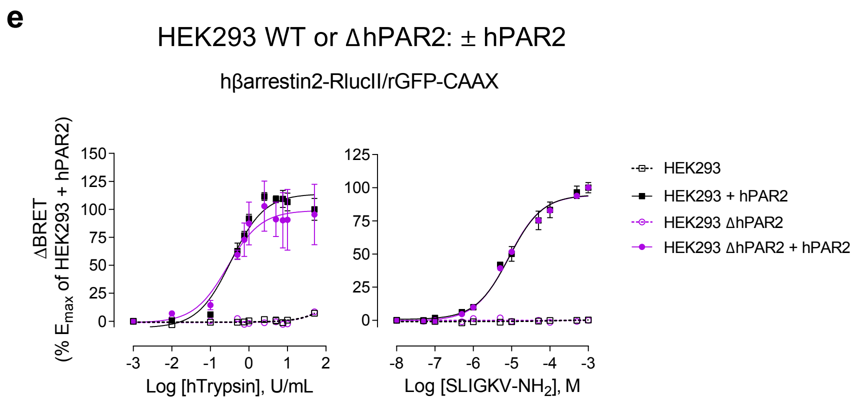

Supplementary Fig. 7: Characterization of βarrestin2 recruitment at the plasma membrane induced by hPAR2 in HEK293 and HCT 116 cells and by mPAR2 in CMT-93 cells. a-c βarrestin2 recruitment to the plasma membrane was measured either in HEK293 (**a**) and HCT 116 (**b**) cells expressing hPAR2 and human ebBRET sensors hβarrestin2-RlucII/rGFP-CAAX after stimulation by hTrypsin or SLIGKV-NH_2 (15 min), or in CMT-93 (**c**) cells expressing mPAR2 with the mouse ebBRET sensor mβarrestin2-RlucII/rGFP-CAAX in response to SLIGRL-NH_2 (15 min). Data presented in **a** are the same as in **Fig. 2g**, but with results expressed as ΔBRET in % E_{max} of hTrypsin for hPAR2 or SLIGRL-NH_2 for mPAR2 (mean ±SEM; n = 3-4). **d** Summary of potencies (pEC_{50}) and relative efficacies (E_{max}) of βarrestin2 recruitment induced by PAR2 ligands. Data are the mean ±SEM deduced from the concentration-response curves above. **e** Dose-response curves of βarrestin2 recruitment induced by increasing concentrations of hTrypsin or SLIGKV-NH_2 (15 min) in WT or hPAR2 knockout (ΔhPAR2) HEK293 cells transfected with control DNA or hPAR2 and ebBRET sensors βarrestin2-RlucII/rGFP-CAAX. Results are expressed as ΔBRET in % E_{max} of HEK293 + hPAR2 cells (mean ±SEM; n = 3-4).

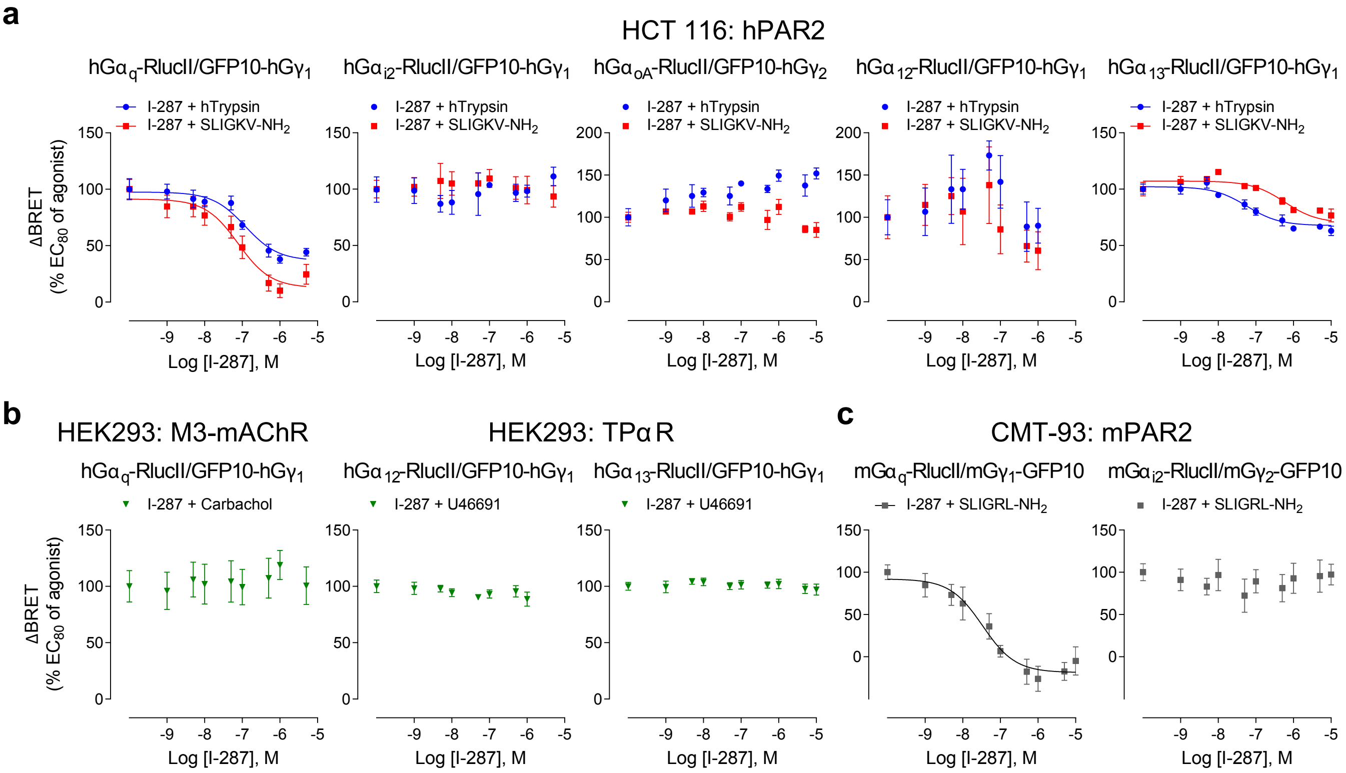

d

Pathway	Treatment	HEK293: hPAR2 pIC50	HEK293: hPAR2 Imax (%)	HCT 116: hPAR2 pIC50	HCT 116: hPAR2 Imax (%)	CMT-93: mPAR2 pIC50	CMT-93: mPAR2 Imax (%)
$G\alpha_q$	I-287 + hTrypsin	-7.14 ± 0.23	47.8 ± 5.6	-6.92 ± 0.17	60.9 ± 5.4	-	-
	I-287 + SLIGKV-NH_2	-7.35 ± 0.32	73 ± 11.6	-7.11 ± 0.19	78.6 ± 7.6	-	-
	I-287 + SLIGRL-NH_2	-	-	-	-	-7.47 ± 0.20	110.7 ± 10.5
$G\alpha_{13}$	I-287 + hTrypsin	-6.41 ± 0.12	49.3 ± 2.6	-7.24 ± 0.21	34.5 ± 3.5	-	-
	I-287 + SLIGKV-NH_2	-6.52 ± 0.14	59.1 ± 3.7	-6.24 ± 0.19	37.2 ± 3.3	-	-

Supplementary Fig. 8: I-287 is a biased antagonist of PAR2-promoted Gα proteins activation. a-c Impact of I-287 on G proteins activation using the BRET²-based α/βγ dissociation biosensors. HCT 116 cells expressing hPAR2 (**a**), HEK293 cells expressing hM3-mAChR (G_q) or hTPαR ($G_{12/13}$) (**b**) and CMT-93 cells expressing mPAR2 (**c**) were co-transfected with the human or mouse biosensors Gα-RlucII/Gγ-GFP10, as indicated. Cells were pretreated with increasing concentrations of I-287 for 15 min and response was measured after stimulation with an EC_{80} concentration of hTrypsin or SLIGKV-NH_2 for hPAR2, SLIGRL-NH_2 for mPAR2, carbachol for M3-mAChR and U46691 for hTPαR. Results are expressed as ΔBRET in % of the response induced by EC_{80} of respective agonists in the absence of I-287 (mean ±SEM; n = 3-7). **d** Summary of potencies (pIC_{50}) and relative inhibition (I_{max}) of I-287 on G proteins activation induced by PAR2 ligands. Data are the mean ± SEM deduced from the concentration-response curves above.

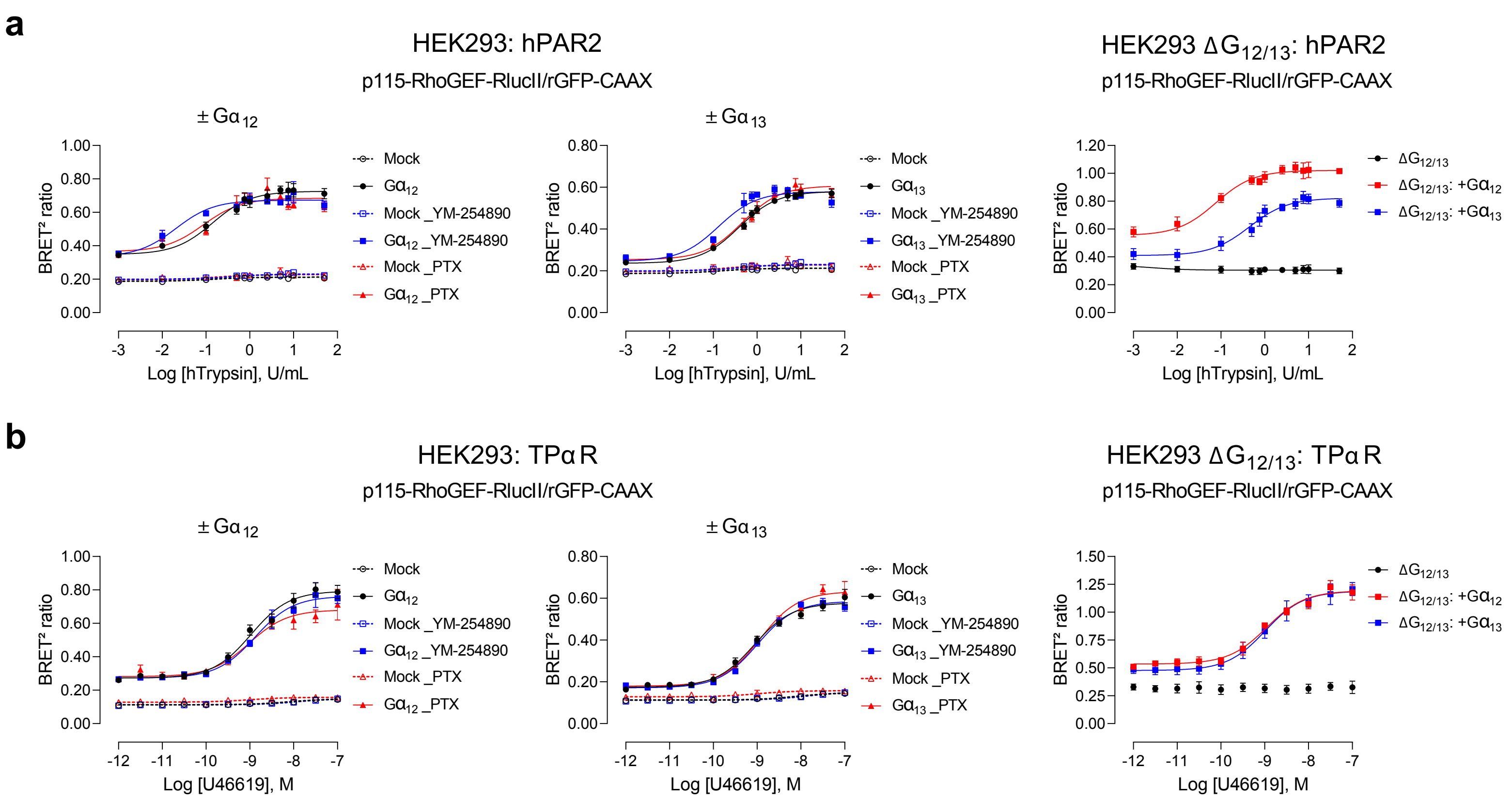

Supplementary Fig. 9: Validation of p115-RhoGEF ebBRET-based sensors to monitor G$\alpha_{12/13}$ **protein activation.** Evaluation of p115-RhoGEF recruitment at the plasma membrane in cells transfected with the hPAR2 (**a**) or TPαR (**b**) receptors and the G$\alpha_{12/13}$ activation sensors (p115-RhoGEF-RlucII/rGFP-CAAX), along with the Gα_{12} or Gα_{13} subunits. Dose-response curves elicited by hTrypsin (1 min, hPAR2; **a**) or U46691 (1 min, TPαR; **b**) stimulation of HEK293 cells expressing control DNA (Mock), Gα_{12} (*left panels*) or Gα_{13} (*central panels*) and pretreated or not with YM-254890 (1 µM, 30 min) or PTX (100 ng/mL, 18 h), or HEK293T G$\alpha_{12/13}$ knockout cells (ΔG$_{12/13}$) supplemented or not with Gα_{12} (ΔG$_{12/13}$: +G$_{12}$) or Gα_{13} (ΔG$_{12/13}$: +G$_{13}$) subunits (*right panels*). Results are expressed as BRET² ratio of absolute values (mean ± SEM; n = 4-8).

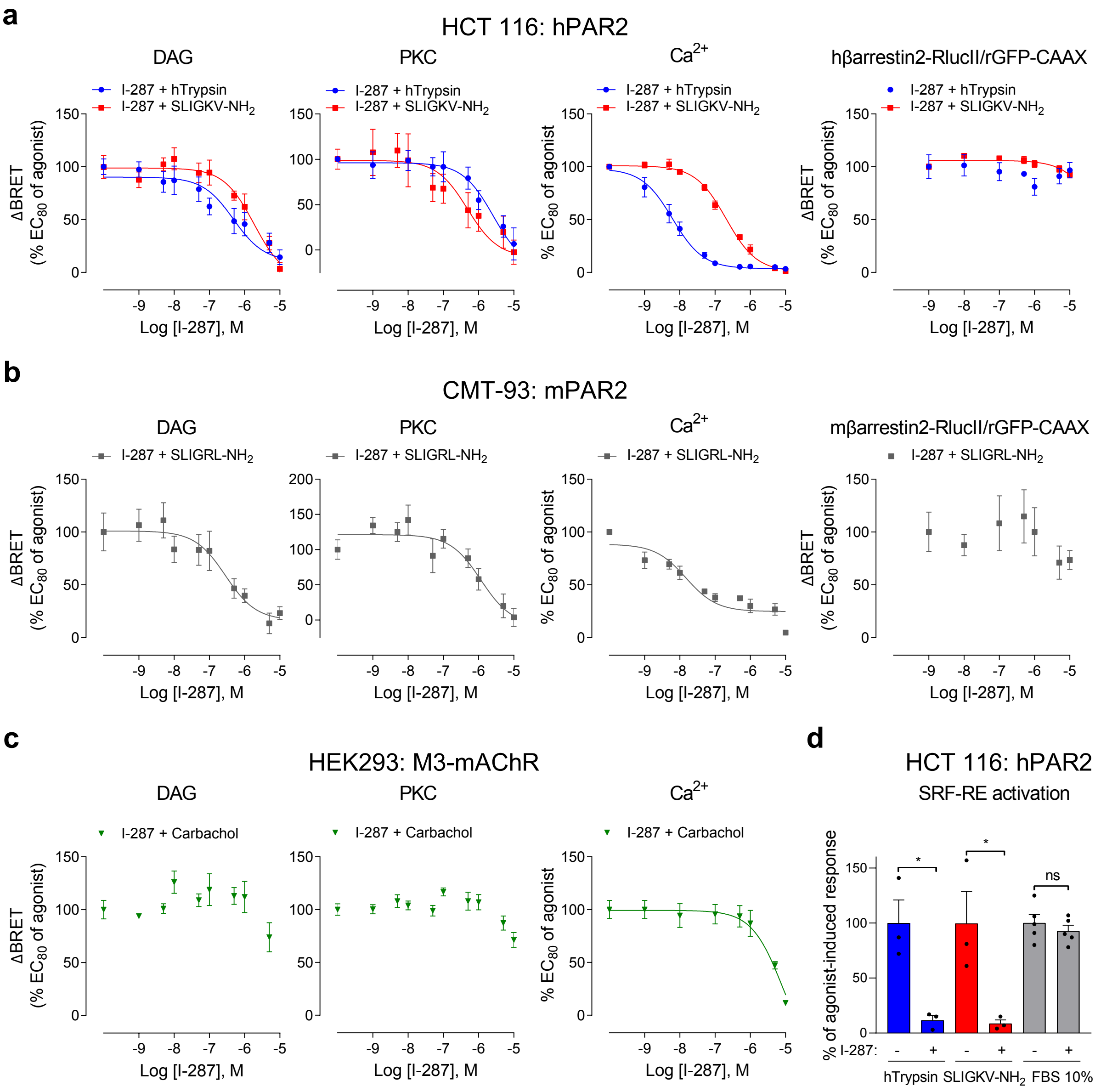

e

Pathway	Treatment	HEK293: hPAR2 pIC50	HEK293: hPAR2 Imax (%)	HCT 116: hPAR2 pIC50	HCT 116: hPAR2 Imax (%)	CMT-93: mPAR2 pIC50	CMT-93: mPAR2 Imax (%)
DAG	I-287 + hTrypsin	-6.69 ± 0.21	108.5 ± 12.0	-6.32 ± 0.18	79.8 ± 6.6	-	-
	I-287 + SLIGKV-NH2	-6.25 ± 0.18	114.5 ± 10.1	-5.73 ± 0.15	106.5 ± 7.5	-	-
	I-287 + SLIGRL-NH2	-	-	-	-	-6.54 ± 0.27	84.5 ± 11.1
PKC	I-287 + hTrypsin	-6.87 ± 0.22	90.9 ± 10.0	-5.59 ± 0.22	116.6 ± 14.8	-	-
	I-287 + SLIGKV-NH2	-6.78 ± 0.15	93.8 ± 6.8	-6.32 ± 0.25	107 ± 12.3	-	-
	I-287 + SLIGRL-NH2	-	-	-	-	-5.93 ± 0.25	128.6 ± 19.4
Ca^{2+}	I-287 + hTrypsin	-7.94 ± 0.08	92.8 ± 4.0	-8.21 ± 0.06	94.7 ± 3.6	-	-
	I-287 + SLIGKV-NH2	-6.86 ± 0.06	97.3 ± 2.8	-6.69 ± 0.04	100.4 ± 1.8	-	-
	I-287 + SLIGRL-NH2	-	-	-	-	-7.83 ± 0.17	63.8 ± 5.5

Supplementary Fig. 10: I-287 selectively inhibits PAR2-mediated activation of DAG/Ca^{2+}/PKC and RhoA signaling pathways but had no effect on βarrestin2 recruitment. a-c Impact of I-287 on DAG/Ca^{2+}/PKC signaling and βarrestin2 recruitment at the plasma membrane promoted by PAR2 or M3-mAChR. For DAG and PKC experiments, HCT 116 (**a**), CMT-93 (**b**) and HEK293 (**c**) cells were co-transfected with hPAR2, mPAR2 or M3-mAChR, respectively, and either the $BRET^2$-based biosensors DAG or PKC. For βarrestin2 recruitment experiments, HCT 116 and CMT-93 cells were co-transfected with hPAR2 (**a**) or mPAR2 (**b**), respectively, and the ebBRET sensors βarrestin2 RlucII/rGFP-CAAX. Cells were pretreated with increasing concentrations of I-287 for 15 min and response was measured after stimulation with an EC_{80} concentration of either hTrypsin or SLIGKV-NH_2 for hPAR2, SLIGRL-NH_2 for mPAR2 or carba-chol for M3-mAChR. All data are expressed as ΔBRET in % of the response induced by EC_{80} of respective agonists in the absence of I-287 (mean ± SEM; n = 3-5). For Ca^{2+} experiments, HCT 116 (**a**), CMT-93 (**b**) and HEK293 (**c**) cells endogenously expressing hPAR2, mPAR2 and M3-mAChR respectively, were pretreated with increasing concentrations of I-287 for 30 min and Ca^{2+} release was measured after stimulation with an EC_{80} concentration of either hTrypsin or SLIGKV-NH_2 for hPAR2, SLIGRL-NH_2 for mPAR2 and carbachol for M3-mAChR. All data are shown as % of the response induced by respective agonists in the absence of I-287 (mean ± SEM; n = 3-4). **d** Impact of I-287 on hPAR2-promoted SRF-RE reporter gene activation induced after 6 h stimulation with hTrypsin (10 U/mL) or SLIGKV-NH_2 (100 µM) in HCT 116 cells expressing hPAR2. FBS (10%) was used as control. Results are expressed as % of the response induced by respective agonists in the absence of I-287 (mean ± SEM; n = 3-5; unpaired t-test: *$p < 0.05$ compared to respective control cells, ns = not-significant). **e** Summary of potencies (pIC_{50}) and relative inhibition (I_{max}) of I-287 on DAG/Ca^{2+}/PKC signaling induced by PAR2 ligands. Data are the mean ± SEM deduced from the concentration response curves above.

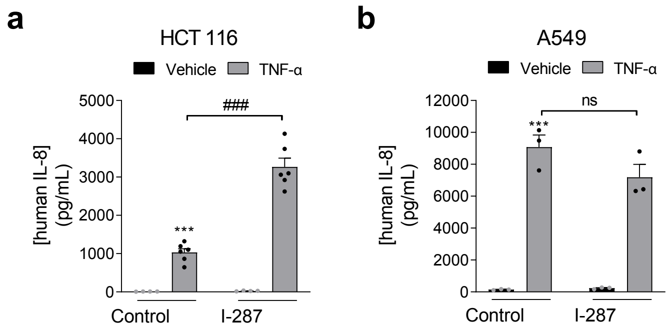

Supplementary Fig. 11: Differential impact of I-287 on TNF-α-induced secretion of IL-8 cytokine. Inhibitory action of I-287 (10 μM, 30 min) on TNF-α promoted IL-8 cytokine release induced after 6 h stimulation with vehicle or TNF-α (10 ng/mL) in culture medium of HCT 116 (**a**) and A549 (**b**) cells expressing hPAR2. Data are expressed as IL-8 concentration in pg/mL (mean ± SEM; n = 3-6; Two-way ANOVA followed by Tukey's post-hoc: ***p < 0.001 compared to control cells with vehicle; $^{###}$p < 0.001 compared to control cells with respective agonist).

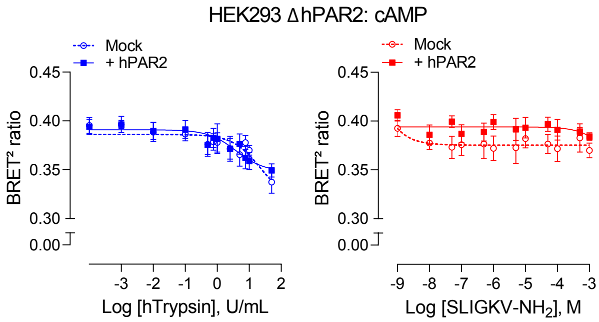

Supplementary Fig. 12: cAMP-elicited by hTrypsin is hPAR2-independent. cAMP production measurement induced by increasing concentrations of hTrypsin or SLIGKV-NH_2 (5 min) in HEK293 cells deleted for hPAR2 and co-expressing the EPAC biosensor in the presence or absence of hPAR2. Results are expressed as BRET² ratio of absolute values (mean ± SEM; n = 4).

Supplementary Fig. 13: Uncropped Immunoblots.

Uncropped immunoblots for Fig. 2i

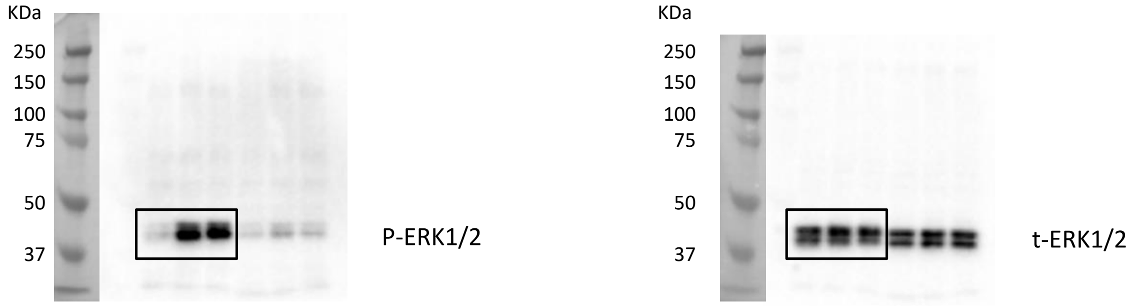

Uncropped immunoblots for Fig. 5e

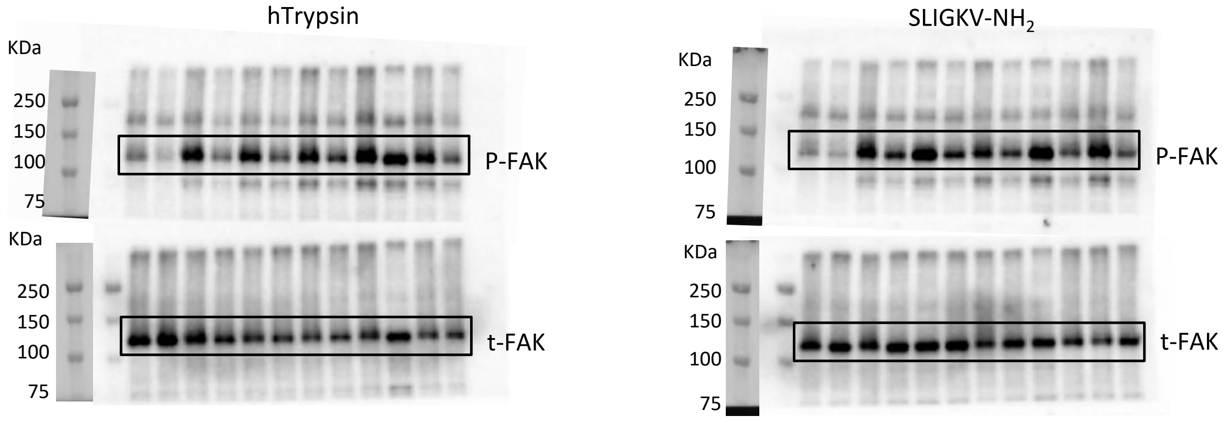

Uncropped immunoblots for Fig. 5f

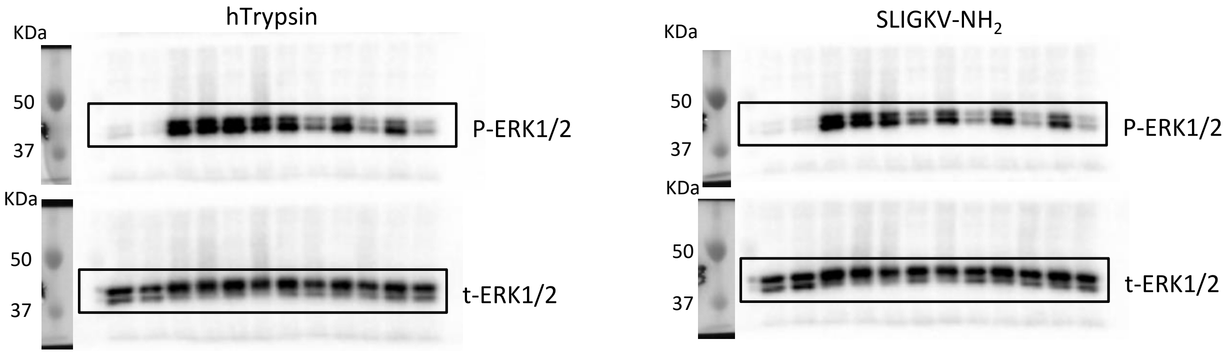

Uncropped immunoblots for Supplementary Fig. 6a and c

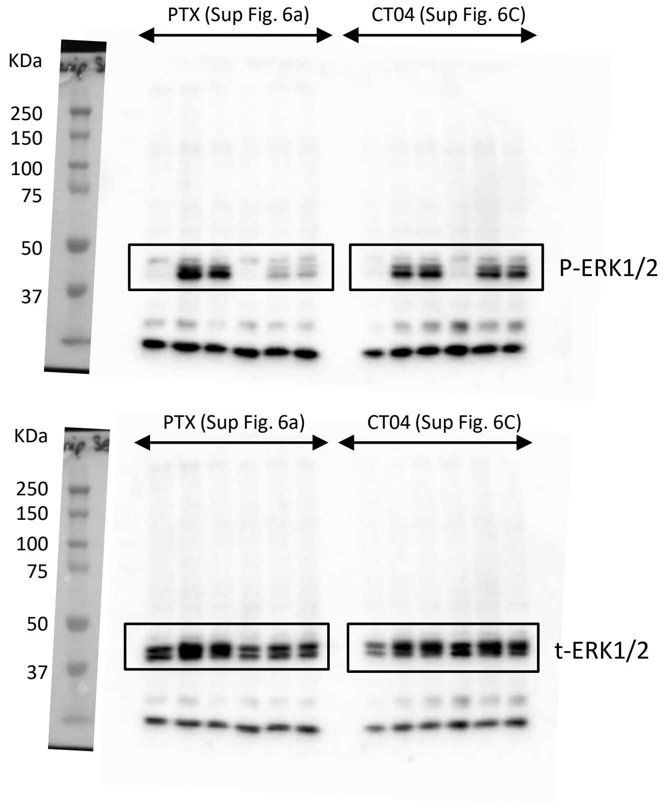

Uncropped immunoblots for Supplementary Fig. 6b

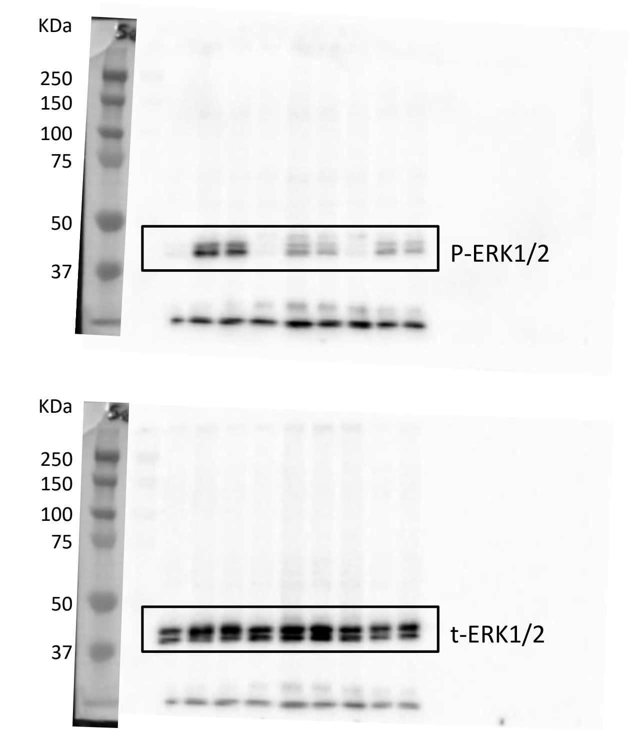

Uncropped immunoblots for Supplementary Fig. 6d

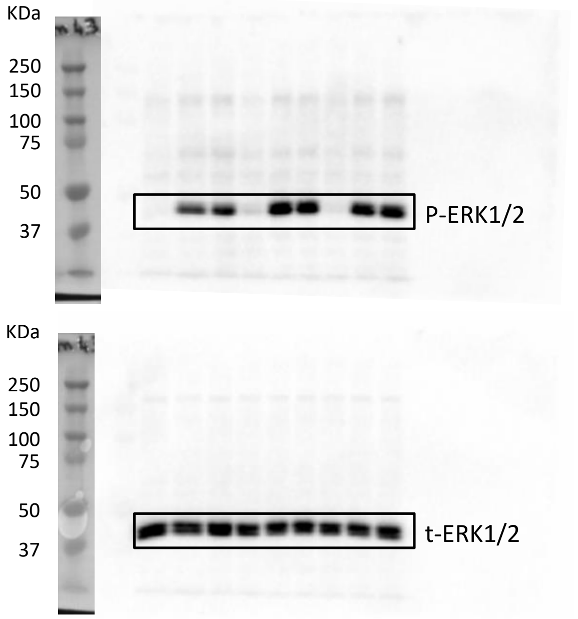

Supplementary Methods

I-287 Compound Synthesis

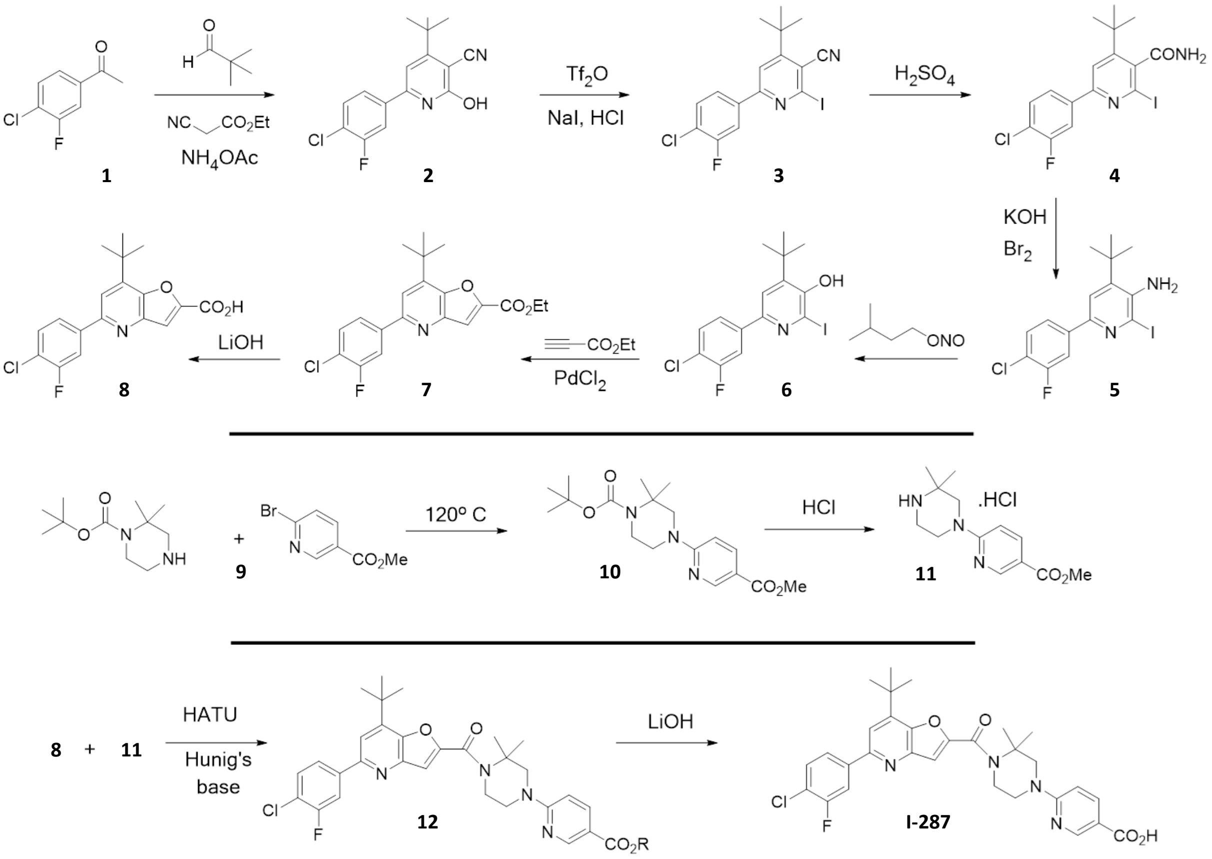

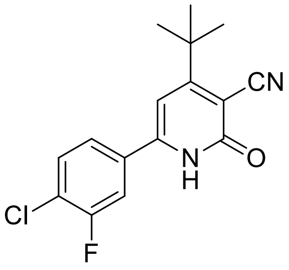

2

4-(tert-butyl)-6-(4-chloro-3-fluorophenyl)-2-oxo-1,2-dihydropyridine-3-carbonitrile [2]

Ammonium Acetate (27.1 g, 352 mmol) was added to a mixture of 1-(4-chloro-3-fluorophenyl)ethanone (8.35 g, 48.4 mmol), pivalaldehyde (6.37 mL, 44.0 mmol) and ethyl 2-cyanoacetate (4.7 mL, 44.0 mmol) in ethanol (29.3 mL) and the resulting mixture was heated at 85°C for 18 h. After cooling to RT, water (15 mL) was added and let stirred 30 min. The solid is filtered and rinse with more water. The wet solid obtained is then stirred in methanol for 15 min, filtered and dried to give 4.3 g (34%) of the title compound. ^{1}H NMR (400 MHz, $CDCl_3$) δ ppm 1.57 (s, 9H) 6.71 (s, 1 H) 7.63 - 7.68 (m, 2 H) 7.70 (d, *J*=1.96 Hz, 1 H) 13.07 (br. s, 1 H).

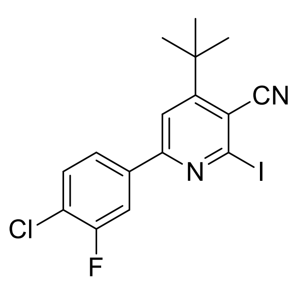

3

4-(tert-butyl)-6-(4-chloro-3-fluorophenyl)-2-iodonicotinonitrile [3]

To a suspension of 4-(tert-butyl)-6-(4-chloro-3-fluorophenyl)-2-oxo-1,2-dihydropyridine-3-carbonitrile (4.3 g, 14.11 mmol) in acetonitrile (141 mL) was added pyridine (1.48 mL, 18.34 mmol). The mixture was brought to 0°C and triflic anhydride (2.98 mL, 17.64 mmol) was added dropwise. The suspension was slowly brought to RT and stirred overnight yielding a clear solution. After cooled at 0°C, sodium iodide (10.58 g, 70.6 mmol) was added followed by the dropwise addition of HCl 4.0 M (4.23 mL, 16.93 mmol) in dioxane. After stirring overnight, it was heated for 40 min at 80°C. The mixture was quenched with sodium bicarbonate, diluted with ethyl acetate and washed with a thiosulfate solution. The aqueous phase was extracted 3 times with ethyl acetate and the combined extract washed with brine. The solution was dried over sodium sulfate, filtered and the solvent removed. Purification on ISCO using a RediSep® column (Hx/EA; 0-20%) gave 1.57 g (27%) of the title compound. ^{1}H NMR (400 MHz, $CDCl_3$) δ ppm 1.57 (s, 9 H) 7.53 (dd, *J*=8.44, 7.46 Hz, 1 H) 7.72 (s, 1 H) 7.76 (ddd, J=8.30, 2.08, 0.86 Hz, 1 H) 7.86 (dd, J=10.03, 2.20 Hz, 1 H). LRMS + H^+= 415.0.

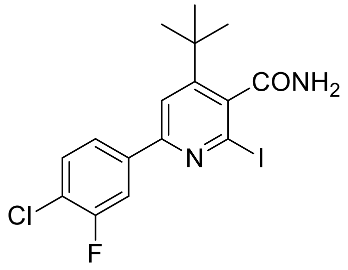

4

4-(tert-butyl)-6-(4-chloro-3-fluorophenyl)-2-iodonicotinamide [4]

A suspension of 4-(tert-butyl)-6-(4-chloro-3-fluorophenyl)-2-iodonicotinamide (1.62 g, 3.9 mmol) and sulfuric acid (4.1 mL) was heated overnight at 85°C. After cooling to RT, it was neutralized with potassium carbonate and then extracted with dichloromethane (2 times). The combined extracts were washed with an ammonium chloride solution dried over sodium sulfate and filtered. The solvent was removed to give 1.62 g (100%) crude of the title compound. ^{1}H NMR (400 MHz, $CDCl_3$) δ ppm 1.50 (s, 9 H) 5.80 (br. s., 1 H) 6.05 (br. s, 1 H) 7.49 (dd, J=8.31, 7.34 Hz, 1 H) 7.64 - 7.73 (m, 2 H) 7.80 (dd, J=10.15, 2.08 Hz, 1 H). LRMS + H^+= 433.0.

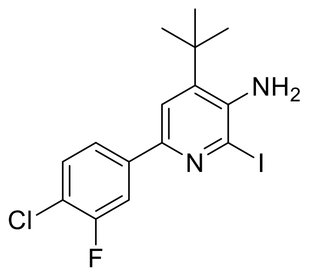

5

4-(tert-butyl)-6-(4-chloro-3-fluorophenyl)-2-iodopyridin-3-amine [5]

Bromine was added to a 0°C solution of KOH (1.15 g) in water (3.3 mL). After 5 min of stirring 4-(tert-butyl)-6-(4-chloro-3-fluorophenyl)-2-iodonicotinamide (1.1 g, 2.54 mmol) was added solid followed 5 min later by the addition of 0.15 mL of water and by 0.15 mL THF. After 15 min, the mixture was brought to RT for 1.5 h. The solution was diluted with ethyl acetate and the organic phase separated. The remaining phase was extracted 2 times with ethyl acetate which were combined and dried over sodium sulfate. The solvent was removed to yield crude 0.98 g (89%) of the title compound. ^{1}H NMR (400 MHz, $CDCl_3$) δ ppm 1.46 (s, 9 H) 4.52 (br. s, 2 H) 7.37 - 7.48 (m, 2 H) 7.58 - 7.65 (m, 1 H) 7.71 (dd, *J*=10.64, 2.08 Hz, 1 H). LRMS + H^+= 405.0.

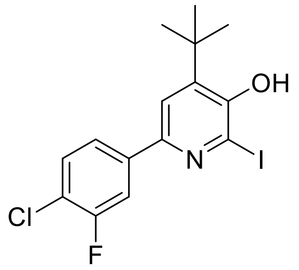

6

4-(tert-butyl)-6-(4-chloro-3-fluorophenyl)-2-iodopyridin-3-ol [6]

Isopentyl nitrite (1.4 mL, 10.9 mmol) was added dropwise to 4-(tert-butyl)-6-(4-chloro-3-fluorophenyl)-2-iodopyridin-3-amine (1.48 g, 3.6 mmol) in 2,2,2-trifluoroacetic acid (6.6 mL, 86 mmol). After 30 min at RT methanol (22.0 mL, 545 mmol) and potassium carbonate (8.80 g, 63.6 mmol) were added. The mixture was stirred for 5 h at the end of which water was added and the pH adjusted to 5 with diluted HCl. After stirring for 5 h it was extracted 3x with ethyl acetate and the combined extracts were washed with brine, dried over sodium sulfate, filtered and the solvent removed. Purification on ISCO using a RediSep® column (Hx/Tol; 0-70%) gave 0.565 g (39%) of the title compound. ^{1}H NMR (400 MHz, $CDCl_3$) δ ppm 1.44 (s, 9 H) 5.66 (s, 1 H) 7.40 - 7.51 (m, 2 H) 7.62 (dd, *J*=2.05, 0.88 Hz, 1 H) 7.74 (dd, *J*=10.52, 2.05 Hz, 1 H). LRMS + H^+= 406.0.

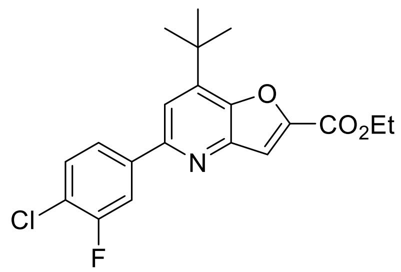

7

Ethyl 7-(tert-butyl)-5-(4-chloro-3-fluorophenyl)furo[3,2-b]pyridine-2-carboxylate [7]

To a degas solution of 4-(tert-butyl)-6-(4-chloro-3-fluorophenyl)-2-iodopyridin-3-ol (0.39 g, 0.96 mmol), ethyl propiolate (0.13 mL, 1.2 mmol) and potassium carbonate (0.399 g, 2.88 mmol) in tetahydrofuran (6.4 mL) was added copper (I) iodide (18 mg, 0.096 mmol) and $PdCl_2(PPh_3)_2$ (27 mg, 0.038 mmol) and this was heated to 75°C for 12 h. The mixture was quenched with an ammonium chloride solution and diluted with ethyl acetate. The organic phase was separated and the aqueous phase extracted 2 times with ethyl acetate. The combined extracts were dried over sodium sulfate, filtered and the solvent removed. Purification on ISCO using a RediSep® column (Hx/EA; 0-10%) gave 0.106 g (29%) of the title compound. ^{1}H NMR (400 MHz, $CDCl_3$) δ ppm 1.47 (t, *J*=7.09 Hz, 3 H) 1.60 (s, 9 H) 4.49 (q, *J*=7.09 Hz, 1 H) 7.52 (dd, *J*=8.31, 7.58 Hz, 1 H) 7.63 (s, 1 H) 7.69 (s, 1 H) 7.76 (ddd, *J*=8.38, 2.02, 0.86 Hz, 1 H) 7.86 (dd, *J*=10.39, 2.08 Hz, 1 H). LRMS + H^+= 376.1.

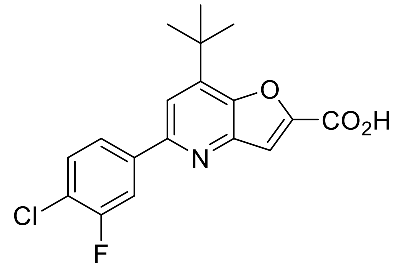

8

7-(tert-butyl)-5-(4-chloro-3-fluorophenyl)furo[3,2-b]pyridine-2-carboxylic acid [8]

LiOH (0.56 mL, 1.12 mmol) was added to a solution of ethyl 7-(tert-butyl)-5-(4-chloro-3-fluorophenyl)furo[3,2-b]pyridine-2-carboxylate (0.211 g, 0.56 mmol) in THF (2.5 mL). Methanol (0.1 mL) was added until one phase was obtained. The solution was heated at 45°C for 1 h, quenched with formic acid and diluted with ethyl acetate. The organic phase was separated and the aqueous phase extracted 2 times with ethyl acetate. The organic phases were combined and washed with a NaCl solution. The organic phase was dried over sodium sulfate, filtered and the solvent removed to give 0.195 g (100%) of the title compound. ^{1}H NMR (400 MHz, DMSO-d_6) δ ppm 1.52 (s, 9 H) 7.66 - 7.74 (m, 1 H) 7.78 (s, 1 H) 7.85 (s, 1 H) 8.02 (dd, *J*=8.41, 1.76 Hz, 1 H) 8.16 (dd, *J*=10.96, 1.96 Hz, 1 H). LRMS + H^+= 348.1.

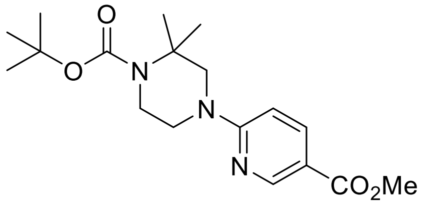

10

tert-butyl 4-(5-(methoxycarbonyl)pyridin-2-yl)-2,2-dimethylpiperazine-1-carboxylate [10]

A mixture of tert-butyl 2,2-dimethylpiperazine-1-carboxylate (0.069 g, 0.32 mmol) and methyl 6-bromonicotinate (0.077 g, 0.35 mmol) in N-methylpyrrolidone was heated at 120°C in a microwave oven for 5 h. The mixture was diluted with water and extracted 2 times with ethyl acetate. The combined organic extracts were washed with a NaCl solution, dried over sodium sulfate and absorbed on SiO_2. Purification on ISCO using a RediSep® column (Hx/EA; 0-30%) gave 0.056 g (50%) of the title compound. ^{1}H NMR (400 MHz, $CDCl_3$) δ ppm 1.42 (s, 6 H) 1.51 (s, 9 H) 3.56 (t, *J*=7.00 Hz, 2 H) 3.86 - 3.94 (m, 7 H) 6.42 (d, *J*=9.29 Hz, 1 H) 8.06 (dd, *J*=9.05, 2.45 Hz, 1 H) 8.80 (dd, *J*=2.45, 0.73 Hz, 1 H). LRMS + H^+= 350.3.

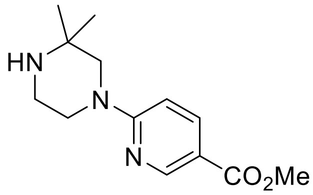

11

methyl 6-(3,3-dimethylpiperazin-1-yl)nicotinate (HCl salt) [11]

HCl (5.19 mL, 20.75 mmol) in 4.0 M in dioxane was added slowly to a solution of tert-butyl 4-(5-(methoxycarbonyl)pyridin-2-yl)-2,2-dimethylpiperazine-1-carboxylate (0.290 g, 0.830 mmol) in dichloromethane (4.1 mL). After 2 h, the solvent was removed and the crude taken in chloroform and the solvent was evaporated again. This was repeated a second time to yield 0.237 g of the title compound. LRMS + H^+= 250.2.

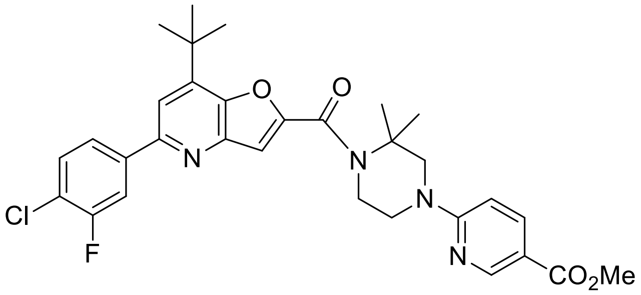

12

methyl 6-(4-(7-(tert-butyl)-5-(4-chloro-3-fluorophenyl)furo[3,2-b]pyridine-2-carbonyl)-3,3-dimethylpiperazin-1-yl)nicotinate [12]

2-(3H-[1,2,3]triazolo[4,5-b]pyridin-3-yl)-1,1,3,3-tetramethylisouronium hexafluorophosphate(V) (0.204 g, 0.53 mmol) was added to a solution of 7-(tert-butyl)-5-(4-chloro-3-fluorophenyl)furo[3,2-b]pyridine-2-carboxylic acid (0.170 g, 0.48 mmol) in DMF (2.4 mL). After 10 min. Hunig's base (0.42 mL, 2.4 mmol) was added followed by methyl 6-(3,3-dimethylpiperazin-1-yl)nicotinate hydrochloride (0.144 g, 0.50 mmol). After 90 min a NH_4Cl solution was added followed by ethyl acetate. The organic phase was separated and the aqueous phase extracted 3 times with ethyl acetate. The combined

organic phase was washed with a $NaHCO_3$ solution followed by water and dried over Na_2SO_4. After filtration, the solvent was removed and the crude residue purification on ISCO using a RediSep® column (Hx/EA; 0-50%) to yield 0.180 g of the title compound. ^1H NMR (400 MHz, $CDCl_3$) δ ppm 1.57 (s, 9 H) 1.66 (s, 6 H) 3.74 (t, J=5.67 Hz, 2 H) 3.88 (s, 3 H) 4.02 (s, 2 H) 4.10 - 4.16 (m, 2 H) 6.46 (d, J=9.00 Hz, 1 H) 7.43 (s, 1 H) 7.50 (t, J=8.02 Hz, 1 H) 7.58 (s, 1 H) 7.70 - 7.78 (m, 1 H) 7.84 (dd, J=10.37, 1.76 Hz, 1 H) 8.09 (dd, J=9.00, 1.96 Hz, 1 H) 8.82 (d, J=2.35 Hz, 1 H). LRMS + H^+= 579.2.

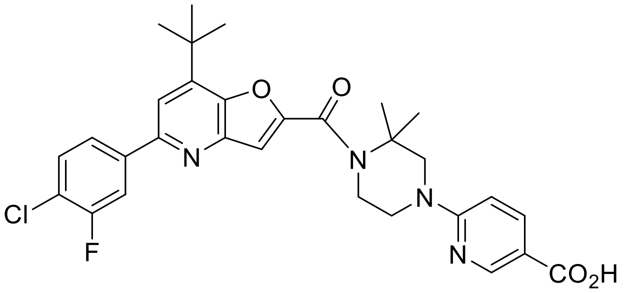

I-287

6-(4-(7-(tert-butyl)-5-(4-chloro-3-fluorophenyl)furo[3,2-b]pyridine-2-carbonyl)-3,3-dimethylpiperazin-1-yl)nicotinic acid [I-287]

LiOH 2.0 M (0.36 mL, 0.72 mmol) was added to a solution of methyl 6-(4-(7-(tert-butyl)-5-(4-chloro-3-fluorophenyl)furo[3,2-b]pyridine-2-carbonyl)-3,3-dimethylpiperazin-1-yl)nicotinate (0.210 g, 0.36 mmol) in THF (1.4 mL) followed by the addition of methanol until one phase was obtained. The solution was then heated at 45°C for 5 h and then diluted with ethyl acetate and water. Formic acid (0.14 mL, 3.63 mmol) was added and the organic phase separated. The aqueous phase was extracted 2 times with ethyl acetate and the combined organic extracts washed with water. The solution was dried over Na_2SO_4, filtered and the solvent removed. Purification on ISCO using a RediSep® column (DCM/EA; 0-50%) gave 0.138 g of the title compound. ^1H NMR (400 MHz, DMSO-d_6) δ ppm 1.54 (s, 6 H) 1.55 (s, 9 H) 3.68 (t, *J*=5.48 Hz, 2 H) 4.02 (s, 2 H) 4.12 (t, *J*=5.28 Hz, 2 H) 6.69 (d, *J*=9.39 Hz, 1 H) 7.56 (s, 1 H) 7.72 (t, *J*=8.02 Hz, 1 H) 7.84 (s, 1 H) 7.98 (dd, *J*=9.00, 2.35 Hz, 1 H) 8.04 (dd, *J*=8.61, 1.96 Hz, 1 H) 8.18 (dd, *J*=10.96, 1.96 Hz, 1 H) 8.65 (d, *J*=2.35 Hz, 1 H) 12.49 (br. s., 1 H). LRMS + H^+= 565.2.

Supplementary References

1. Mende, F. et al. Translating biased signaling in the ghrelin receptor system into differential in vivo functions. Proc Natl Acad Sci U S A 115, E10255-E10264, doi:10.1073/pnas.1804003115 (2018).
2. Gales, C. et al. Probing the activation-promoted structural rearrangements in preassembled receptor-G protein complexes. Nat Struct Mol Biol 13, 778-786, doi:10.1038/nsmb1134 (2006).
3. Quoyer, J. et al. Pepducin targeting the C-X-C chemokine receptor type 4 acts as a biased agonist favoring activation of the inhibitory G protein. Proc Natl Acad Sci U S A 110, E5088-5097, doi:10.1073/pnas.1312515110 (2013).
4. Ehrlich, A. T. et al. Biased Signaling of the Mu Opioid Receptor Revealed in Native Neurons. iScience 14, 47-57, doi:10.1016/j.isci.2019.03.011 (2019).
5. Namkung, Y. et al. Functional selectivity profiling of the angiotensin II type 1 receptor using pathway-wide BRET signaling sensors. Sci Signal 11, doi:10.1126/scisignal.aat1631 (2018).
6. Leduc, M. et al. Functional selectivity of natural and synthetic prostaglandin EP4 receptor ligands. J Pharmacol Exp Ther 331, 297-307, doi:10.1124/jpet.109.156398 (2009).
7. Namkung, Y. et al. Monitoring G protein-coupled receptor and beta-arrestin trafficking in live cells using enhanced bystander BRET. Nat Commun 7, 12178, doi:10.1038/ncomms12178 (2016).
