# Supplementary material for: The PAR2 inhibitor I-287 selectively targets Gαq and Gα12/13 signaling and has anti-inflammatory effects
Source: Commun Biol. 2020 Nov 27;3:719. doi: 10.1038/s42003-020-01453-8 (PMC7695697; doi:10.1038/s42003-020-01453-8)
Supplement: Supplementary file 2 — Reporting Summary [file 42003_2020_1453_MOESM2_ESM.pdf]

nature research

Corresponding author(s): Michel Bouvier

Last updated by author(s): Nov 17, 2020

Reporting Summary

Nature Research wishes to improve the reproducibility of the work that we publish. This form provides structure for consistency and transparency in reporting. For further information on Nature Research policies, see our Editorial Policies and the Editorial Policy Checklist.

Statistics

For all statistical analyses, confirm that the following items are present in the figure legend, table legend, main text, or Methods section.

n/a	Confirmed	
☐	☒	The exact sample size (*n*) for each experimental group/condition, given as a discrete number and unit of measurement
☐	☒	A statement on whether measurements were taken from distinct samples or whether the same sample was measured repeatedly
☐	☒	The statistical test(s) used AND whether they are one- or two-sided<br>*Only common tests should be described solely by name; describe more complex techniques in the Methods section.*
☒	☐	A description of all covariates tested
☒	☐	A description of any assumptions or corrections, such as tests of normality and adjustment for multiple comparisons
☐	☒	A full description of the statistical parameters including central tendency (e.g. means) or other basic estimates (e.g. regression coefficient) AND variation (e.g. standard deviation) or associated estimates of uncertainty (e.g. confidence intervals)
☒	☐	For null hypothesis testing, the test statistic (e.g. *F*, *t*, *r*) with confidence intervals, effect sizes, degrees of freedom and *P* value noted<br>*Give P values as exact values whenever suitable.*
☒	☐	For Bayesian analysis, information on the choice of priors and Markov chain Monte Carlo settings
☒	☐	For hierarchical and complex designs, identification of the appropriate level for tests and full reporting of outcomes
☒	☐	Estimates of effect sizes (e.g. Cohen's *d*, Pearson's *r*), indicating how they were calculated

Our web collection on statistics for biologists contains articles on many of the points above.

Software and code

Policy information about availability of computer code

Data collection	BRET, fluorescence and luminescence were measured on TriStar² LB 942 Multimode Microplate Reader (Berthold Technologies), FlexStation II microplate reader (Molecular Devices) and SpectraMax L (Molecular Devices), respectively.<br>Western blots detection was performed using a ChemiDoc MP Imaging System (BioRad).
Data analysis	In vitro and in vivo assays were analysed using GraphPad Prism. Relative densitometry analysis on protein bands for western blots was performed using MultiGauge software (Fujifilm).

For manuscripts utilizing custom algorithms or software that are central to the research but not yet described in published literature, software must be made available to editors and reviewers. We strongly encourage code deposition in a community repository (e.g. GitHub). See the Nature Research guidelines for submitting code & software for further information.

Data

Policy information about availability of data

All manuscripts must include a data availability statement. This statement should provide the following information, where applicable:

- Accession codes, unique identifiers, or web links for publicly available datasets
- A list of figures that have associated raw data
- A description of any restrictions on data availability

The authors declare that all data supporting the findings in this study are presented within the article and its Supplementary Information files. The data that support the findings of this study are available from the corresponding author upon reasonable request.

Field-specific reporting

Please select the one below that is the best fit for your research. If you are not sure, read the appropriate sections before making your selection.

☒ Life sciences ☐ Behavioural & social sciences ☐ Ecological, evolutionary & environmental sciences

For a reference copy of the document with all sections, see nature.com/documents/nr-reporting-summary-flat.pdf

Life sciences study design

All studies must disclose on these points even when the disclosure is negative.

Sample size	For in vitro experiments, data were repeated independently at least three (3) times.<br>For in vivo experiments, no specific statistical method was used to calculate sample size. The sample size selected was based on previous experiences with the same assays and are in line with the state of the art for similar assays.
Data exclusions	Some data were excluded for Fig 2e (calcium experiments) because of drug distribution problems by the instrument. Also, some values are absent (Fig 2k and 5e) from certain replicates because these concentrations were not tested.
Replication	For all cellular assays the experiments were repeated at least three times on different days.<br>For the animal studies, the individual experiments were repeated on different days and were reproducible.
Randomization	For cellular experiments, the cells used for the studies came from the same pool of cells that were then distrubuted randmomly in the different experimental groups. Similarly, the mice used in the studies came from the same colony and were split randomly between the experimental groups.
Blinding	The investigators were blinded to group allocation during compounds administration to mice in the in vivo experiments.

Reporting for specific materials, systems and methods

We require information from authors about some types of materials, experimental systems and methods used in many studies. Here, indicate whether each material, system or method listed is relevant to your study. If you are not sure if a list item applies to your research, read the appropriate section before selecting a response.

Materials & experimental systems

n/a	Involved in the study	
☐	☒	Antibodies
☐	☒	Eukaryotic cell lines
☒	☐	Palaeontology and archaeology
☐	☒	Animals and other organisms
☒	☐	Human research participants
☒	☐	Clinical data
☒	☐	Dual use research of concern

Methods

n/a	Involved in the study	
☒	☐	ChIP-seq
☒	☐	Flow cytometry
☒	☐	MRI-based neuroimaging

Antibodies

Antibodies used	Rabbit Anti-FAK, phospho (Tyr397) Monoclonal Antibody, Unconjugated, Clone EP2160Y (P-FAK; Abcam; catalog no: ab81298, 1:1000 dilution; RRID:AB_1640500),<br>FAK antibody [EP695Y](Abcam; catalog no: ab40794, 1:1000 dilution; RRID:AB_732300),<br>p44/42 MAP kinase (phosphorylated Erk1/2) antibody (P-ERK1/2; Cell Signaling Technology; catalog no. 9101; 1:1000 dilution; RRID:AB_331646),<br>p44/42 MAPK (Erk1/2) Antibody (total ERK1/2; Cell Signaling Technology; catalog no. 9102; 1:1000 dilution; RRID:AB_330744),<br>anti-Rabbit IgG, peroxidase-linked species-specific whole antibody (from donkey) Secondary Antibody (GE Healthcare; catalog no, NA934 1:3000 (ERK) or 1:5000 (FAK) dilutions; RRID:AB_772206).<br>Antibodies used to performed "Investigation of IL-8 Release" were from the DuoSet ELISA human CXCL8/IL-8 immunoassay kit (R&D Systems).
Validation	P-FAK: validation and references available on Abcam website (https://www.abcam.com/fak-phospho-y397-antibody-ep2160y-ab81298.html)<br>Total-FAK: validation and references available on Abcam website (https://www.abcam.com/fak-antibody-ep695y-ab40794.html)<br>P-ERK1/2: validation and references available on Cell Signaling website (https://www.cellsignal.com/products/primary-antibodies/phospho-p44-42-mapk-erk1-2-thr202-tyr204-antibody/9101)<br>Total-ERK1/2: validation and references available on Cell Signaling website (https://www.cellsignal.com/products/primary-antibodies/p44-42-mapk-erk1-2-antibody/9102?site-search-type=Products&N=4294956287&Ntt=9102%3B&fromPage=plp&_requestid=5467677).

DuoSet ELISA human CXCL8/IL-8 immunoassay kit: validation and references available on R&D Systems website (https://www.rndsystems.com/products/human-il-8-cxcl8-duoset-elisa_dy208).

Eukaryotic cell lines

Policy information about cell lines

Cell line source(s)	Human embryonic kidney 293 (HEK293), mouse rectal carcinoma (CMT-93), human colon carcinoma (HCT 116) and human lung carcinoma (A549) cells were obtained from the American Type Culture Collection.<br>HEK293 cells devoid of functional Gα12 and Gα13 proteins (ΔG12/G13), a gift from A. Inoue (Tohoku University, Sendai, Miyagi, Japan), were previously described (Devost, D. et al. Biol Chem 2017; Namkung, Y. et al. Sci Signal 2018).<br>HEK293 cells devoid of functional βarrestin1/2 by the CRISPR/Cas9 system (Δβarrestin1/2), a gift from S. Laporte (McGill University, Montreal, Quebec, Canada), were previously described (Luttrell, L. M. et al. Sci Signal 2018).<br>HEK293 cells devoid of functional hPAR2 have been generated by the CRISPR/Cas9 system by Vertex Pharmaceuticals (Canada) Inc.
Authentication	Since these cells are routinely used by the investigators, only visual authentication based on the morphology of the cells was used .
Mycoplasma contamination	All cell lines tested negative for mycoplasm
Commonly misidentified lines<br>(See ICLAC register)	*Name any commonly misidentified cell lines used in the study and provide a rationale for their use.*

Animals and other organisms

Policy information about studies involving animals; ARRIVE guidelines recommended for reporting animal research

Laboratory animals	Adult male C57BL/6J mice weighing 25-30 g (Charles River laboratories, Canada)
Wild animals	Study did not involve wild animals
Field-collected samples	Study did not involve samples collected from the field
Ethics oversight	All procedures were performed in accordance with the Canadian Council on Animal Care (CCAC) and with the International Association for the Study of Pain (IASP) guidelines for pain research on animals. Procedures were also approved by the local Animal Care Committee at the Université de Sherbrooke and were part of protocol 242-14.

Note that full information on the approval of the study protocol must also be provided in the manuscript.
